# L1-ORF1p nucleoprotein can rapidly assume distinct conformations and simultaneously bind more than one nucleic acid

**DOI:** 10.1093/nar/gkae1141

**Published:** 2024-11-20

**Authors:** Ben A Cashen, M Nabuan Naufer, Michael Morse, Micah J McCauley, Ioulia Rouzina, Charles E Jones, Anthony V Furano, Mark C Williams

**Affiliations:** Northeastern University, Department of Physics, 360 Huntington Avenue, Boston, MA 02115, USA; Northeastern University, Department of Physics, 360 Huntington Avenue, Boston, MA 02115, USA; Northeastern University, Department of Physics, 360 Huntington Avenue, Boston, MA 02115, USA; Northeastern University, Department of Physics, 360 Huntington Avenue, Boston, MA 02115, USA; Ohio State University, Department of Chemistry and Biochemistry, Center for Retroviral Research and Center for RNA Biology, 281 W Lane Avenue, Columbus, OH 43210, USA; The Laboratory of Molecular and Cellular Biology, NIDDK, NIH, 8 Center Drive, Bethesda, MD 20892, USA; The Laboratory of Molecular and Cellular Biology, NIDDK, NIH, 8 Center Drive, Bethesda, MD 20892, USA; Northeastern University, Department of Physics, 360 Huntington Avenue, Boston, MA 02115, USA

## Abstract

LINE-1 (L1) is a parasitic retrotransposable DNA element, active in primates for the last 80–120 Myr. L1 has generated nearly one-third of the human genome by copying its transcripts, and those of other genetic elements (e.g. Alu and SVA), into genomic DNA by target site-primed reverse transcription (TPRT) and remains active in modern humans. L1 encodes two proteins that bind their encoding transcript (*cis* preference) to form an L1 ribonucleoprotein (RNP) that mediates retrotransposition. ORF2p provides reverse transcriptase and endonuclease activity. ORF1p, its major component, is a homo-trimeric phospho-protein that binds single-stranded nucleic acid (ssNA) with high affinity and exhibits nucleic acid (NA) chaperone activity. We used optical tweezers to examine ORF1p binding to individual single-stranded DNA (ssDNA) molecules and found that the arrangement of ORF1p on the ssDNA depends on their molar ratio. When the concentration of ORF1p is just sufficient to saturate the entire NA molecule, the nucleoprotein (NP) is compact and stable. However, additional ORF1p binds and destabilizes the compacted NP, allowing it to engage a second ssDNA. Our results suggest that ORF1p displaced from its RNA template during TPRT could bind and destabilize remaining downstream L1 RNP, making them susceptible to hijacking by non-L1 templates, and thereby enable retrotransposition of non-L1 transcripts.

## Graphical Abstract

**Graphical Abstract:**
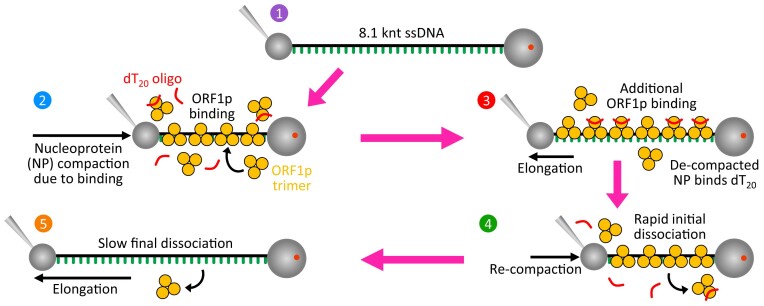

## Introduction

**Figure 1.**
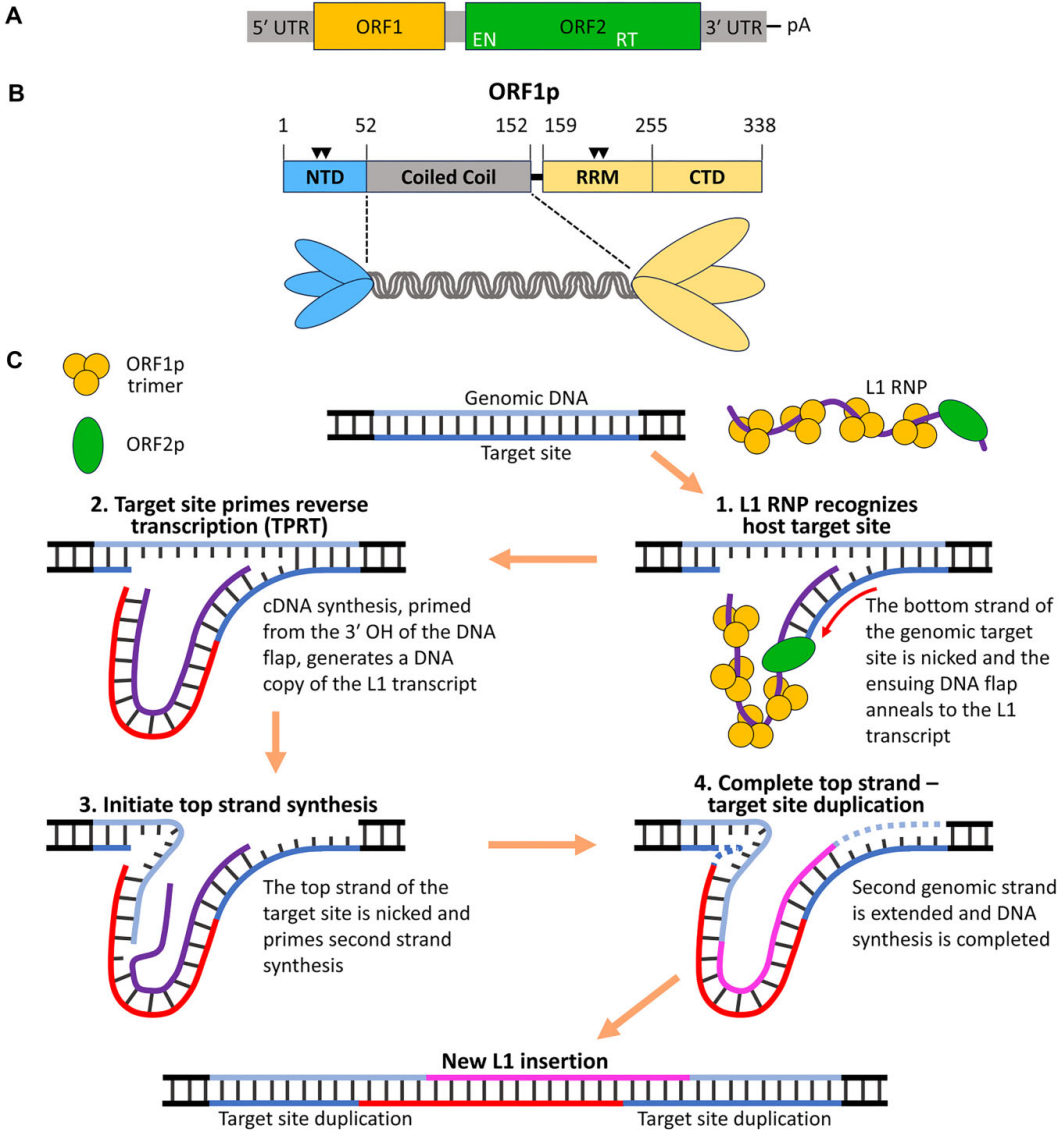
L1 and ORF1p. (**A**) Domain organization of a typical full-length human L1 element. Positions of the 5′ untranslated region (UTR), open reading frame 1 (ORF1), open reading frame 2 [ORF2, including endonuclease (EN) and reverse transcriptase (RT) domains], 3′ UTR and poly A tail are shown. (**B**) Domain structure and trimeric depiction of ORF1p. Amino acid positions of the N-terminal domain (NTD), coiled coil (CC), RNA recognition motif (RRM) and C-terminal domain (CTD). Approximate locations of the four phosphorylation sites are denoted by black triangles. (**C**) Diagram illustrating the steps of target site-primed reverse transcription (TPRT). The bottom strand of the genomic target site (blue) is nicked by ORF2p EN and the 3′ end of the L1 RNA (purple) is annealed to the DNA flap to generate a primer for complementary DNA (cDNA) synthesis (1.). The L1 transcript is reverse transcribed by ORF2p RT to synthesize the first L1 cDNA (red) (2.). The top strand of the target site (light blue) is nicked and subsequently annealed to the cDNA, priming synthesis of the second L1 strand (3.). The second genomic strand (pink) is extended, and L1 DNA synthesis is completed (4.). The newly inserted L1 element is flanked by target site duplications.

LINE-1 (L1, Long INterspersed Element-1) is a non-Long Terminal Repeat (LTR) retrotransposon that has been replicating and evolving in mammals for ∼100 Myr. L1 replicates through a ‘copy-paste’ mechanism whereby its transcript (*cis* preference) is copied into genomic DNA (retrotransposition, Figure 1). L1 is the only active autonomous mobile element in humans (1–4) and has generated ∼17% of the human genome. Despite strong *cis* preference, it can also retrotranspose non-L1 transcripts, such as those produced from processed pseudogenes and non-autonomous transposable elements (e.g. Alu and SVA), accounting for about 13% of human DNA (5–9). Consequently, L1 activity has produced as much as ∼30% of the mass of some mammalian genomes, contributing to genetic expansion, alteration, and rearrangements. Strong negative selection (10) and a number of host repressive mechanisms (e.g. APOBEC cytosine deamination and L1 DNA methylation) (11) indicate that L1 is a harmful parasite.

Nearly all vertebrates contain L1 elements (generally ≥ 6 kb) that share the same structural motifs (Figure 1A): a 5′ UTR, which has regulatory function, two protein encoding sequences (ORF1 and ORF2), and a 3′ UTR that in mammals contains conserved G-rich sequences capable of forming non-B DNA structures that may stimulate retrotransposition (12–15), terminating in a 3′ A-rich sequence. Figure 1B depicts the major structural motifs of ORF1p. All ORF1ps contain a CC domain (16), which in mouse and human was shown to mediate trimerization of the ORF1p monomer (16–20). ORF1p trimers are the major component of the L1 ribonucleoprotein (L1 RNP; Figure 1C), which also includes ORF2p and mediates L1 replication (21–24). This occurs by TPRT, which takes place *in situ* at 5′(T)_n_(A)_n_3′/3′(A)_n_(T)_n_5′ sites of the host genome, catalyzed by the EN and RT domains of ORF2p: The EN nicks the bottom strand of host DNA at the TA/AT junction, generating a 3′-OH terminated (T)_n_ flap that anneals to the terminal A-rich 3′ sequence of the L1 transcript and primes reverse transcription by the RT domain (15,25–29).

ORF1p is not homologous to any protein of known structure, and the mechanistic details of how ORF1p supports L1 replication are unknown. The protein contains four domains (Figure 1B): an intrinsically disordered NTD that contains two conserved phosphorylation sites required for retrotransposition (30,31), a 14-heptad CC that mediates ORF1p trimerization (16–20), a non-canonical RRM (32), and a carboxy-terminal domain (CTD). Residues within the RRM and CTD endow ORF1p with non-specific, high affinity single-stranded nucleic acid (ssNA) binding and nucleic acid (NA) chaperone activity (facilitates annealing and exchange of NA strands) (33–35).

The biochemical features that underlie NA chaperone activity, e.g., charge neutralization, higher affinity for ssNA than double-stranded (ds) NA, and the ability to lower the cooperativity of the helix-coil transition are properties of the trimer and are balanced to promote both melting and annealing of NA (14,15,17,18,20,22,32–39). Mutations in the RRM or CTD domains that eliminate NA chaperone activity also abolish retrotransposition, consistent with a role for ORF1p chaperone activity in retrotransposition (33). Subsequent studies showed that ORF1p can promote and stabilize hybridization of mismatched duplexes (17), which are likely to be encountered during hybridization of the target site DNA and L1 transcript to generate a productive primer for cDNA synthesis by ORF2p.

Retrotransposition *in vivo* is correlated with the ability of ORF1p to form stable NA-bound ORF1p polymers *in vitro* (40,41), likely mediated by inter-protein contacts between amino acid residues in the C-terminal half of the trimer (i.e. distinct from CC-mediated trimerization) (17). Various single amino acid substitutions in the CC can abolish both *in vitro* polymer formation on NA and retrotransposition (40,41), demonstrating the sensitivity of ORF1p function to its CC sequence (42).

ORF1p must be efficiently displaced from its template during TPRT to ensure cDNA synthesis by ORF2p (Figure 1C). Therefore, ORF1p has the seemingly paradoxical capabilities to strongly bind and compact the ssNA template while also allowing its efficient release during cDNA synthesis. This could be achieved by a lower affinity of ORF1p for dsNA than ssNA, and we previously showed that ORF1p binds dsNA with about 1/8^th^ the affinity of ssNA (17). Using force-melted (overstretched) λ phage DNA as a substrate for ORF1p binding, we also showed that reducing tension on the ORF1p-bound NA allowed duplex formation by unwound portions of the NA. This process displaced a fraction of stably bound ORF1p (41), analogous to dissolution of the L1 RNP during TPRT.

**Figure 2.**
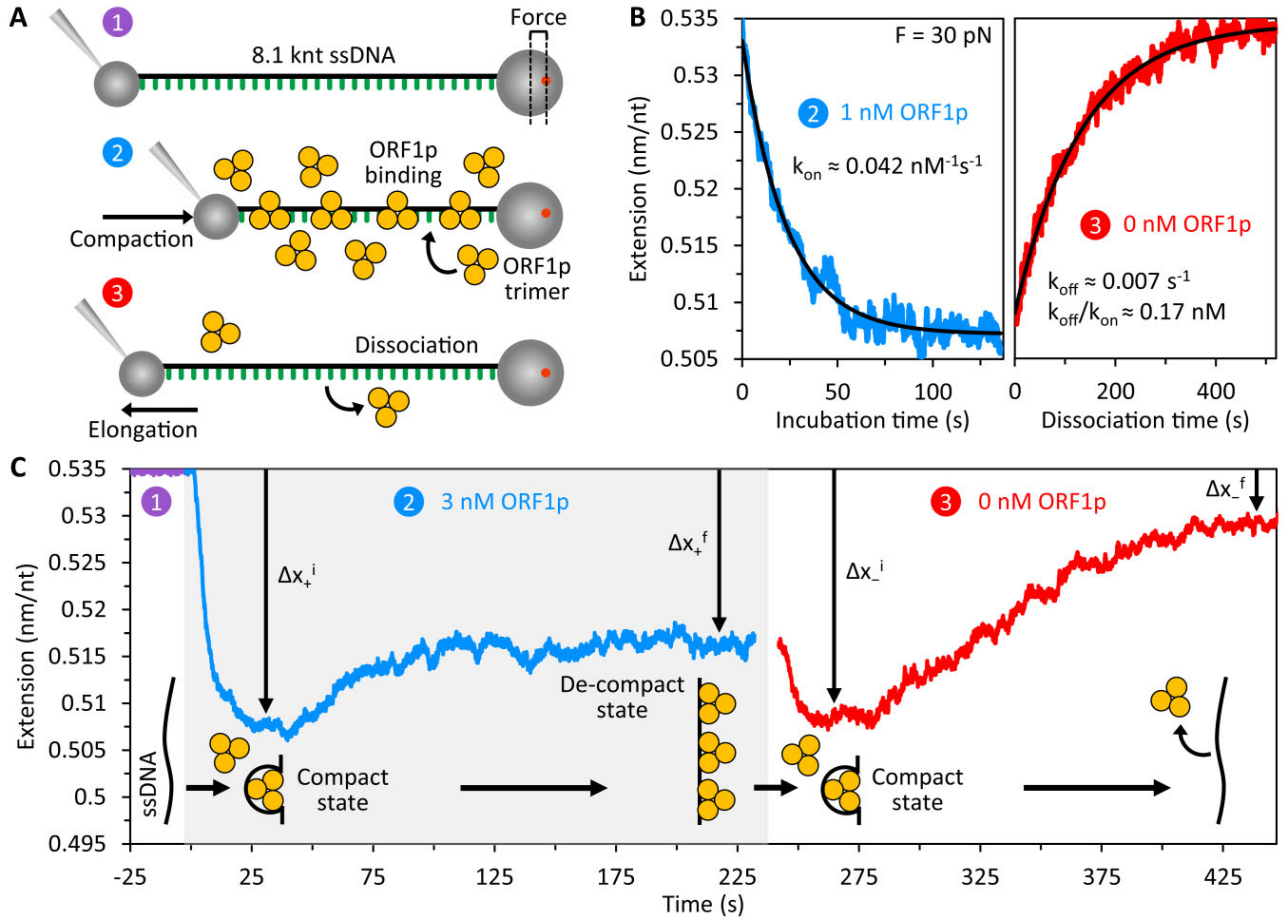
Measuring ORF1p binding and ssDNA conformation. (**A**) An 8.1 knt ssDNA was tethered between two functionalized microbeads and held at a fixed tension as measured by laser deflection of the optical trap (1.). Extension of the DNA was continuously adjusted to maintain constant tension during incubation with free protein and its subsequent removal to measure ORF1p binding (2.) and dissociation (3.). (**B**) At a fixed force of 30 pN, incubation with 1 nM ORF1p results in monotonic DNA compaction (left). Upon removal of ORF1p from solution, compaction is completely reversed (right) as the protein dissociates, returning the DNA to its protein-free conformation. The on and off rates are calculated from exponential fits to the data, yielding k_off_/k_on_ ≈ 0.17 nM at 30 pN. (**C**) Extension of the ssDNA in the presence of higher ORF1p concentrations (3 nM) reveals two binding phases: an initial rapid compaction (Δx_+_^i^) followed by a slower partial elongation (Δx_+_^f^). Upon removal of protein from solution, two corresponding dissociation steps are observed. Initial dissociation results in fast re-compaction of the substrate (Δx_−_^i^). Subsequent dissociation, marked by a slow increase in ssDNA extension (Δx_−_^f^), is incomplete under these conditions, leaving the strand partially compacted and protein-bound.

Here, we simplified our approach by generating an 8.1 knt ssDNA molecule *in situ*, and employed optical tweezers to examine ORF1p binding (Figure 2A) to individual ssDNA molecules held at different tensions using previously described methods (40,43). Note that NA stretching in optical tweezers typically requires DNA rather than RNA, as the initial tethering process necessitates a stiffer double-stranded substrate. However, the biophysical polymer properties of single-stranded RNA (ssRNA) and ssDNA are nearly identical (44,45), and oligonucleotide-based binding assays (17,35,46) revealed similar non-specific binding affinities of ORF1p (K_D_ ∼ 0.1–1 nM) for both ssDNA and ssRNA, indicating that ssDNA is an appropriate substrate for examining ORF1p–ssNA interactions. We evaluated binding over a wide range of trimer concentrations (1 to 90 nM) and DNA tensions (10 to 50 pN). We also determined the effect of oligo dT_10-60_ on the interaction of ORF1p with the 8.1 knt ssDNA and analyzed binding of a polymerization-deficient ORF1p CC variant, 151p (40,41), as well as an ORF1p monomer, m128p (17), which lacks the NTD and all but the last 3.5 heptads of the CC.

In summary, our single molecule studies show that purified trimeric ORF1ps assemble on an 8.1 knt ssDNA, producing ORF1p nucleoproteins (NPs) that are both dynamic and capacious. The trimers can convert between different binding arrangements as a function of ORF1p concentration and DNA tension. Furthermore, the binding of additional ORF1p to the saturated protein–DNA complex produces a less compact and less stable NP that can accommodate more than one NA substrate. We discuss how an analogous process could occur *in vivo* during TPRT, which would explain why almost half (∼43%) of the genomic DNA generated by L1 activity has resulted from the retrotransposition of non-L1 sequences.

## Materials and methods

### Purification of ORF1p

N-terminal His-tagged ORF1ps were expressed in insect cells and purified as previously described (17).

### Optical tweezers system for measuring ssDNA conformation

An 8.1 knt ssDNA molecule, tethered between two 1.76 μm diameter streptavidin-coated beads, was generated *in situ* (43) and held at various tensions (10–50 pN). Extension of the ssDNA was continuously adjusted to maintain the given force applied by the trapping laser. Unless otherwise stated, experiments were performed in 50 mM Na^+^ (45 mM NaCl and 5 mM NaOH), 10 mM HEPES (2-[4-(2-hydroxyethyl)piperazin-1-yl]ethanesulfonic acid), pH 7.5 (binding buffer) at 21°C (room temperature). Extension of the ssDNA was controlled by a piezoelectric translation stage with 1 nm precision, and the tension along the substrate was measured by laser deflection of the stationary optical trap. The inter-bead distance was measured using bright-field images to determine the absolute extension of the ssDNA and correct for long-term thermal drift in the system. Dissociation was measured after exchanging the protein solution with protein-free buffer following a constant incubation time of 100 s.

### Data analysis and extraction of rates and amplitudes

For constant force experiments, data from individual measurements (ssDNA extension over time) were split into multiple phases in which the DNA extension was either monotonically increasing or decreasing. Each phase was fit to an exponential decay function with two free parameters, an amplitude of extension change at equilibrium (units of nm/nt) and a rate constant (units of s^−1^) (47–49). All data were analyzed using custom scripts in MATLAB (Mathworks) with uncertainty calculated as standard error of the mean (SEM) of three or more replicates.

### Binding of monomeric m128p

Equilibrium extension of the m128p-ssDNA complex was measured as a function of force by first incubating the 8.1 knt ssDNA at 60 pN with 30 nM m128p in binding buffer. At this concentration and temperature (21°C), m128p (comprising the CTD, RRM and 3.5 of the 14 CC heptads) is primarily monomeric (17). Upon reaching binding equilibrium, the extension of the protein–DNA complex was slowly lowered at a rate of ∼10 nm/s to ensure equilibration at every force. The resulting force–extension curve (FEC) was binned with respect to force (1 pN increments), and the FEC of bare ssDNA was subtracted from this data to obtain the change in ssDNA extension associated with m128p binding. These stretching data were compared with measurements of equilibrium extension change from constant force (10, 30 and 50 pN) experiments with 30 nM m128p. Uncertainty in the reported data was calculated as the SEM of three replicate curves.

### ORF1p binding in the presence of dT_n_ oligos

ORF1p was incubated with equimolar dT_n_ (n = 10, 20, 40 and 60) oligos in binding buffer and then incubated with the 8.1 knt ssDNA held at different tensions. Dissociation was measured after replacing the protein-dT_n_ solution with protein-free buffer (with or without dT_n_). Uncertainty in the reported data was calculated as the SEM of three or more replicates.

### Visualizing ORF1p binding to ssDNA

Confocal images were collected with a LUMICKS C-trap. An 8.1 knt ssDNA molecule, tethered between two 3.13 μm diameter streptavidin-coated beads, was generated *in situ* within a parallel-channel laminar flow cell, as described previously (43). Tension on the substrate was measured by beam deflection of the optical trap, and the inter-bead distance was determined by bright-field video analysis. A 488 nm excitation laser was fixed at 1.5 μW, and confocal images were collected at a pixel resolution of 40 nm. The 8.1 knt ssDNA was exposed to a solution of dT_20_ (1 and 30 nM), 5′ labeled with Alexa488 (Integrated DNA Technologies), and to equimolar (1 and 30 nM) solutions of ORF1p (trimer and monomer). Photon counts were displayed and scaled in the Lakeview analysis program (LUMICKS). A constant upper intensity cutoff limited the large autofluorescence from the trapped beads, enabling observation across different conditions. Typical images are shown in Figure 6. Total fluorescence between the beads was integrated and corrected for background (FIJI) for the quantitative results shown in Figure 6H. Intensities were normalized with respect to the highest measured integrated fluorescence signal. Uncertainty was calculated as the SEM of three or more replicates.

## Results

Figure 2B (left) shows the effect of 1 nM wild-type (WT) ORF1p (111p) on the length of an 8.1 knt ssDNA molecule held at 30 pN tension. These conditions produce a single phase of DNA compaction (blue) that is reversed when free protein is removed (red). In contrast, binding at 3 nM (Figure 2C, blue) is biphasic, indicating a more complicated process than a simple one-step binding mechanism. In particular, if each ORF1p bound ssDNA in an identical fashion, shortening would be proportional to the number of proteins bound and the DNA length would decrease monotonically to an asymptote when all ssDNA binding sites are occupied. Instead, the initial ssDNA shortening (Δx_+_^i^) is followed by a slower secondary partial elongation step as the protein–DNA complex equilibrates to a final extension (Δx_+_^f^), greater than that at 1 nM ORF1p. Upon removal of free protein (red), the extension change is similarly biphasic; the ssDNA rapidly shortens (Δx_−_^i^) and returns to its initial compacted length before slowly extending to its protein-free conformation (Δx_−_^f^). Thus, ORF1 proteins can assume multiple conformations on ssDNA, which we term ‘compact’ (maximizes ssDNA shortening) and ‘de-compact’ (minimizes ssDNA shortening).

The protein–DNA state is sensitive to ORF1p concentration and can exhibit a multiphasic binding profile, a phenomenon that we have observed with other single-stranded binding proteins (47–49). When bulk protein exceeds the amount required to saturate the ssNA, the length of the protein-NA complex attains a minimal value (initial compaction) and subsequently elongates as excess ORF1p binds to the ORF1p-ssDNA complex. We surmise that the initial binding phase (Δx_+_^i^) corresponds to ORF1p compacting the ssDNA such that contact between the protein's binding surface and the NA is maximized (see Figure 2C). Subsequent lengthening of the protein-NA complex (Δx_+_^f^) suggests that excess protein competes with and displaces previously bound (fully compacted) ORF1p trimers, which partially disengage from the DNA. Thus, a fraction of the nucleotides that were engaged with ORF1p in its fully compact mode may be transferred to the binding surface of another trimer.

We depict ORF1p dissociation as a reversal of this process—conversion of the de-compacted NP to a fully compacted state—which is facilitated by dissociation of excess ORF1p. More specifically, nucleotides released by departing proteins are incorporated into the binding sites of remaining, partially engaged ssDNA-bound trimers, enabling their transition to a fully compacted conformation (Figure 2C). Their subsequent dissociation in ORF1p-free buffer increases the length of the ssNA as it approaches its protein-free conformation, indicating full release from the DNA molecule. We explored these phenomena as a function of bulk protein concentration and DNA tension by measuring the amplitude and rate associated with each phase of DNA shortening and elongation.

### Concentration dependence of ORF1p binding modes

**Figure 3.**
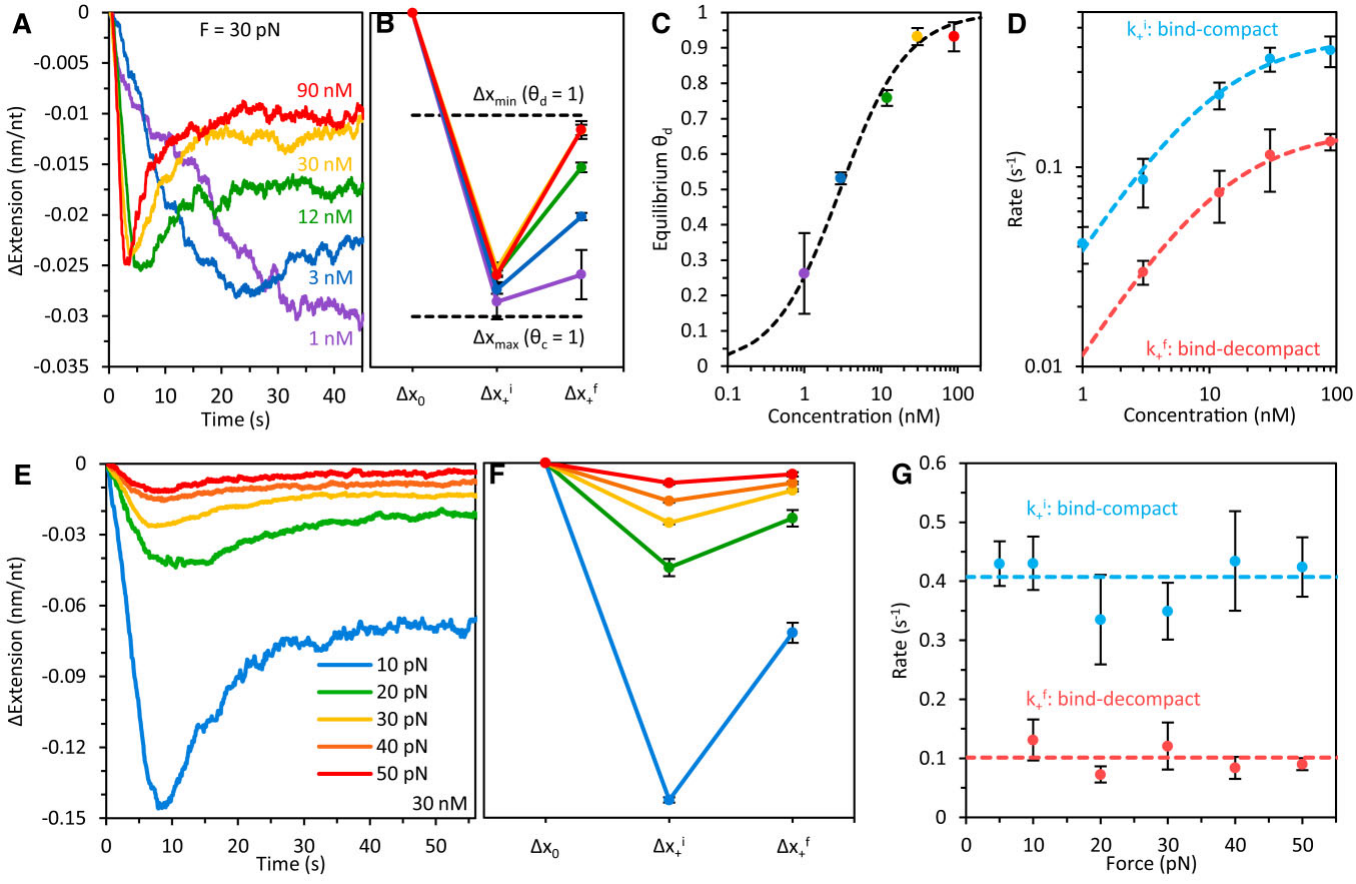
Concentration and force dependence of ORF1p binding phases. (**A**) Representative curves and (**B**) average extension changes associated with ORF1p binding as a function of protein concentration at 30 pN. The secondary partial elongation of ssDNA is more pronounced at higher ORF1p concentrations, and the equilibrium extension reduction of the ssDNA is minimized (consistent with all bound protein assuming a de-compacted state, θ_d_). At low protein concentrations, the reduction in ssDNA extension is maximized (consistent with all bound protein assuming a compact conformation, θ_c_). (**C**) Extension changes of the ssDNA at binding equilibrium are converted to fractional occupancies of ssDNA-bound ORF1p trimers in the de-compacted state (θ_d_) at various concentrations. These data follow the shape of a standard binding isotherm (dashed line) where the two states (θ_d_ and θ_c_) are equally occupied at ∼3 nM ORF1p. (**D**) The rates of each binding step initially increase linearly with protein concentration before reaching an asymptote at high [ORF1p]. (**E**) Representative 30 nM ORF1p binding curves and (**F**) average extension changes at varying ssDNA tensions. Both the initial compaction (Δx_+_^i^) and equilibrium compaction (Δx_+_^f^) of the complex increase as the force is lowered. (**G**) At 30 nM ORF1p, the rates associated with each binding phase are independent of the force applied on the DNA.

Figure 3A–D and Supplementary Table S1 show the effects of different ORF1p concentrations on the length (i.e. extension) of ssDNA held at 30 pN. At this tension, ssDNA is effectively straightened, and its extension is approximately equal to the length of its backbone (i.e. contour length). As excess ORF1p over saturating amounts is increased, the biphasic character of ORF1p binding becomes more pronounced and the final equilibrium compaction of the protein–DNA complex (Δx_+_^f^) decreases (Figure 3A and B), suggesting that high concentrations of free protein favor the formation of less compact ORF1p binding conformers.

Figure 3B shows the average extension changes associated with each binding step for different concentrations of ORF1p, where Δx_max_ denotes the length change of the maximally compacted ORF1p NP state, θ_c_. When [ORF1p] is just above saturating (∼1 nM, purple), this conformation is stable, and the ssDNA remains in its highly compacted state (i.e. a second phase of elongation does not occur). However, at higher concentrations, the ORF1p NP lengthens as it transitions from maximally compacted to partially de-compacted, where Δx_min_ corresponds to the equilibrium length change of the fully de-compacted state, θ_d_. Therefore, at intermediate concentrations, compacted and de-compacted conformations of the ORF1p NPs co-exist.

We converted the equilibrium extension changes (Δx_+_^f^) of the protein–DNA complex to fractional occupancies of ssDNA-bound ORF1p trimers in the de-compacted state, θ_d_ [where θ_d_ = (Δx_max_ – Δx_+_^f^)/(Δx_max_ – Δx_min_), Figure 3C]. θ_d_ follows the shape of a standard two-state binding isotherm (Langmuir model) (50) where both binding states are equally occupied at ∼3 nM ORF1p. At bulk protein concentrations ≥ 30 nM, the fraction of ORF1 proteins in θ_d_ approaches saturation. Therefore, we suggest that transition of the protein-ssNA complex to its de-compacted state (θ_c_ → θ_d_) is facilitated by the binding of additional ORF1p.

Binding kinetics were evaluated by measuring the transition rates associated with each step of DNA compaction (k_+_^i^) and elongation (k_+_^f^) (Figure 3D). The rate of DNA compaction initially increases linearly with concentration before reaching an asymptote at high [ORF1p], as we previously observed with other ssDNA-compacting proteins (47–49). The linearity of k_+_^i^ implies that at low protein concentrations, substrate compaction is rate-limited by the bimolecular association of free protein with the DNA (k_b_ = 0.042 ± 0.006 nM^−1^s^−1^, defined by the slope of the linear k_+_^i^ versus [ORF1p] region). However, at high concentrations, k_+_^i^ plateaus to a constant value, reflecting the fundamental rate of ssDNA compaction by ORF1p (k_c_ = 0.45 ± 0.05 s^−1^) at 30 pN. The DNA elongation rate (k_+_^f^) exhibits a comparable increase with protein concentration, suggesting that this transition phase (de-compaction) involves similar bimolecular binding of the protein to the DNA. However, this process is ∼4-fold slower than initial compaction, indicating an additional (rate-altering) transition step in pre-equilibrium to binding, which may reflect partial ssDNA release from the ORF1p binding site to accommodate additional protein into the complex (see Figure 2C). The high concentration asymptote of k_+_^f^ (k_−c_ = 0.15 ± 0.01 s^−1^) represents the fundamental rate of NP reorganization upon additional ORF1p binding.

### Force dependence of ORF1p binding modes

We also examined ORF1p binding as a function of substrate tension, a critical parameter that both governs formation and reveals properties of the ORF1p-NA complex (Figure 3E–G and Supplementary Table S2). At 30 nM ORF1p, increasing tension (from 10 to 50 pN) reduces both the initial shortening (Δx_+_^i^) and equilibrium shortening (Δx_+_^f^) of the complex by an order of magnitude, implying that ssDNA under high tension resists compaction by ORF1p. Thus, force alters the binding options available to the protein, with high force disfavoring the highly compacted NP states. Nonetheless, at these forces, the binding profiles remain biphasic, indicating transition between the compact and de-compact protein conformations over time. At lower tension (5 pN), however, we did not observe an elongation step subsequent to initial DNA contraction (40), indicating that the highly compacted ORF1p NP which formed at this force is energetically favorable and stable, even at high protein concentrations.

Generally, any process that compacts DNA (e.g. binding of compacting proteins) (47) will be slowed by an applied force, while any process that elongates DNA (e.g. intercalation) (51) will be accelerated. When protein binding produces a single NP conformer, force dependence typically arises from a characteristic length change to the transition state (Δx^†^) associated with each binding event, which when multiplied by the applied force, shifts the free energy barrier, and modulates the force-dependent rate as


(1) \begin{equation*}k(F) = {{k}_0}{{e}^{F \cdot \Delta {{x}^\dag }/{{k}_B}T}}\end{equation*}

However, the rates of compaction and elongation (de-compaction) associated with ORF1p binding (k_+_^i^ and k_+_^f^) are largely insensitive to the tension on the ssDNA substrate (Figure 3G), suggesting that the rate-limiting steps to these binding transitions do not involve significant shortening or lengthening of the DNA molecule (i.e. are not associated with a physical displacement), which would otherwise give rise to force-dependent kinetics.

### Binding of oligomerization-deficient 151p

We previously found that the retrotransposition-null CC ORF1p mutant, 151p, exhibits the same binding and chaperone activity with oligonucleotides N_15-120_ as the retrotransposition-competent WT 111p (41). However, whereas adjacent 111p trimers bound to ssλ phage DNA can form tightly bound, stable oligomers that resist dissociation, 151p trimers are unable to do so (40,41). Formation of stable protein oligomers likely facilitates cooperative binding of ORF1p to ssNA, and previous chemical cross-linking studies showed that contacts between amino acid residues in the carboxy-terminal half of ORF1p mediate their oligomerization (17). Thus, only the particular orientation of the C-terminal halves of adjacent NA-bound trimers that renders them cross-linkable is compatible with retrotransposition, and this orientation is likely determined by the CC sequence (see the ‘Discussion’ section).

Supplementary Figure S1 and Supplementary Table S3 indicate that biphasic binding is not a result of ORF1p oligomerization, as both proteins exhibit similar biphasic extension changes, in extent and rate with 8.1 knt ssDNA. However, when free protein is removed, 151p dissociates faster and more completely, likely due to the lack of stable inter-trimer interactions between NA-bound proteins. Nevertheless, the overall similarity in binding profiles indicates that ORF1p–ORF1p interactions do not significantly alter the extension of the DNA at moderate and high force, consistent with the idea that biphasic binding is primarily driven by changes in protein density on ssDNA.

### Time dependence of ORF1p dissociation

As with binding, ORF1p dissociation occurs in two phases (Figure 2C, red): Upon removal of free protein, the ssDNA immediately shortens (re-compaction, Δx_−_^i^), suggesting that as ORF1p dissociates, the remaining ssDNA-bound proteins transition to a more compact conformation. Thus, nucleotides freed from departing proteins are incorporated into the existing NP, enabling maximum engagement between NA and the ORF1p binding surface. At this point, the protein–DNA complex is in its most compact conformation, similar to the initial binding state (Δx_+_^i^) attained at 3 nM ORF1p (Figure 2C, blue). Final dissociation of ORF1p produces monotonic ssDNA elongation (Δx_−_^f^) as the DNA approaches its protein-free length, suggesting that the remaining DNA-bound proteins cannot incorporate additional nucleotides released by dissociated proteins.

Both the rate and extent of ORF1p dissociation can be affected by its incubation conditions. We had previously shown that a fraction of the ORF1p NP resists dissociation due to protein–protein interactions that can be captured by chemical cross-linking (40,41). Thus, measurements of ORF1p dissociation can report on the oligomeric nature and overall stability of the complex under different conditions. Here, we determined the effect of incubation time on the stability of the NP after removal of free ORF1p. The rate of ssDNA re-compaction (initial shortening, k_−_^i^) decreases significantly with incubation time (Supplementary Figure S2 and Supplementary Table S4), indicating that ORF1p becomes more stably bound to ssDNA, a consequence of increased protein oligomerization. However, regardless of incubation time, the ORF1p-ssDNA complex attains the same maximally compact state (θ_c_), suggesting that the fraction of ORF1p that unbinds during this process is largely independent of the degree of protein oligomerization. Thus, it appears that interactions between DNA-bound ORF1p trimers slow, but do not completely suppress, this transition (θ_d_ → θ_c_) (Supplementary Figure S2A). The aforementioned chemical cross-linking studies (17) showed the residues that mediate inter-trimer interactions differ from those responsible for NA binding activity and revealed that ORF1p oligomers have a higher affinity for DNA than solitary trimers.

The time-dependent ssDNA re-compaction rates (Supplementary Figure S2B) yield an ORF1p oligomerization timescale of τ_oligo_(30 pN) ≈ 100 s, an order of magnitude slower than the biphasic transitions observed during binding. k_−_^i^(t = 0) = 0.11 ± 0.03 s^−1^ defines the rate of initial dissociation in the absence of oligomerization, which is consistent with the re-compaction rate of the oligomerization-deficient 151p (Supplementary Figure S1G). Additionally, k_−_^i^(t → ∞) = 0.010 ± 0.004 s^−1^ indicates that in the limit of long incubation time, trimer–trimer interactions reduce the rate of protein dissociation by ∼10-fold.

### Concentration dependence of ORF1p dissociation

**Figure 4.**
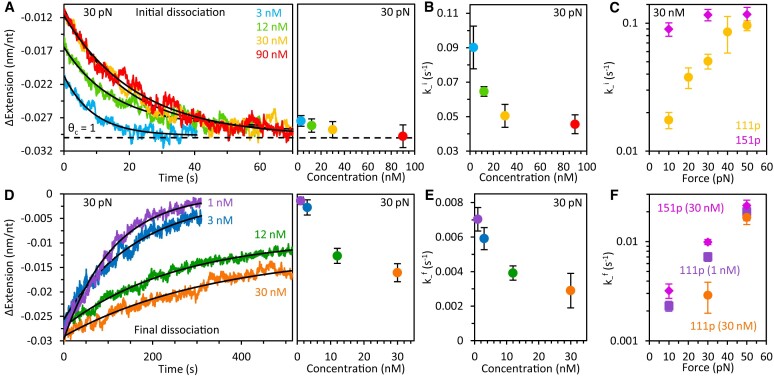
Concentration and force dependence of ORF1p dissociation phases. (**A**) Representative curves (left) and average extension changes (right) of ssDNA during initial dissociation of ORF1p as a function of incubation concentration (see Figure 2C for full trace of dissociation). All data were obtained at 30 pN tension with a constant incubation time of 100 s. Regardless of initial protein concentration, the ORF1p-ssDNA complex attains a similar maximal extension reduction (θ_c_, dashed line). (**B**) The initial dissociation rate (k_−_^i^) decreases with protein concentration. (**C**) The initial dissociation rate of WT ORF1p (circles) increases with ssDNA tension and approaches the force-independent dissociation rate of 151p (diamonds). (**D**) Representative curves (left) and average extension changes (right) of ssDNA during final dissociation of ORF1p as a function of incubation concentration. As concentration is increased, the amount of protein that fully dissociates from the ssDNA decreases and the DNA remains more compact. (**E**) The final dissociation rate (k_−_^f^) decreases with protein concentration. (**F**) Following incubation with 1 nM WT ORF1p (squares), the rate of final dissociation is marginally slower than that of 151p (diamonds), increasing with template tension. Following incubation with 30 nM WT (circles), k_−_^f^ is significantly slower at moderate force, but converges with the dissociation rate of 151p at high force. Note, for 30 nM WT ORF1p, a direct rate could not be measured at 10 pN due to negligible dissociation over the timescale of the experiment (hundreds of seconds).

We further examined ORF1p dissociation by monitoring ssDNA re-compaction (initial dissociation) and elongation (final dissociation) following its incubation with various saturating protein concentrations. Regardless of concentration, the ORF1p-ssDNA complex attains its fully compacted state (θ_c_) (Figure 4A and Supplementary Table S5), consistent with the dependence of Δx_−_^i^ on incubation time (Supplementary Figure S2A). However, the rate of re-compaction (k_−_^i^) is reduced ∼2-fold (Figure 4B), indicating increased NP stability at higher bulk protein concentrations, likely a result of increased oligomerization of NA-bound ORF1p.

Both the rate (k_−_^f^) and extent (Δx_−_^f^) of final dissociation decrease with protein concentration (Figure 4D and E). Comparable to the initial dissociation phase, k_−_^f^ is reduced ∼2-fold over the range of concentrations studied, indicative of increased oligomerization at higher [ORF1p]. However, in contrast to re-compaction, the fraction of ORF1p that unbinds during this dissociation step is reduced and the complex remains more compact (i.e. the ssDNA does not completely return to its protein-free conformation). Additionally, the two phases of dissociation occur over significantly different timescales; k_−_^f^ is more than an order of magnitude (∼15-fold) slower than k_−_^i^, indicating that the highly compacted ORF1p NPs are significantly more resistant to dissociation than the de-compacted binding conformers.

### Force dependence of ORF1p dissociation

The re-compaction rate of WT ORF1p (Figure 4C, k_−_^i^, yellow circles) increases with template tension and approaches the force-independent initial dissociation rate (∼0.1 s^−1^) typical of the non-interacting ORF1p–151 trimers (151p, Figure 4C, magenta diamonds). The relative force response of both proteins indicates that the WT dependence is primarily a consequence of changes in protein oligomerization, suggesting that DNA under high tension prevents ORF1p from forming stable inter-trimeric contacts, resulting in faster protein unbinding and subsequent ssDNA reorganization.

Following incubation with low concentrations of WT ORF1p (1 nM), the rate of final dissociation (Figure 4F, purple squares) is only marginally slower than that of the oligomerization-deficient 151p (Figure 4F, magenta diamonds), increasing strongly (∼10-fold) with template tension (Supplementary Table S6). In contrast, at high WT concentrations (30 nM), k_−_^f^ (Figure 4F, orange circles) is significantly slower at moderate force, corroborating our earlier evidence that increases in concentration may stabilize ORF1p binding by facilitating trimer–trimer interactions on the ssDNA. Regardless of initial protein concentration, however, the rates of WT dissociation converge with the dissociation rate of 151p at high force (50 pN), further supporting the idea that tension on DNA progressively inhibits the formation of stable ORF1p oligomers.

### Binding of monomeric m128p

**Figure 5.**
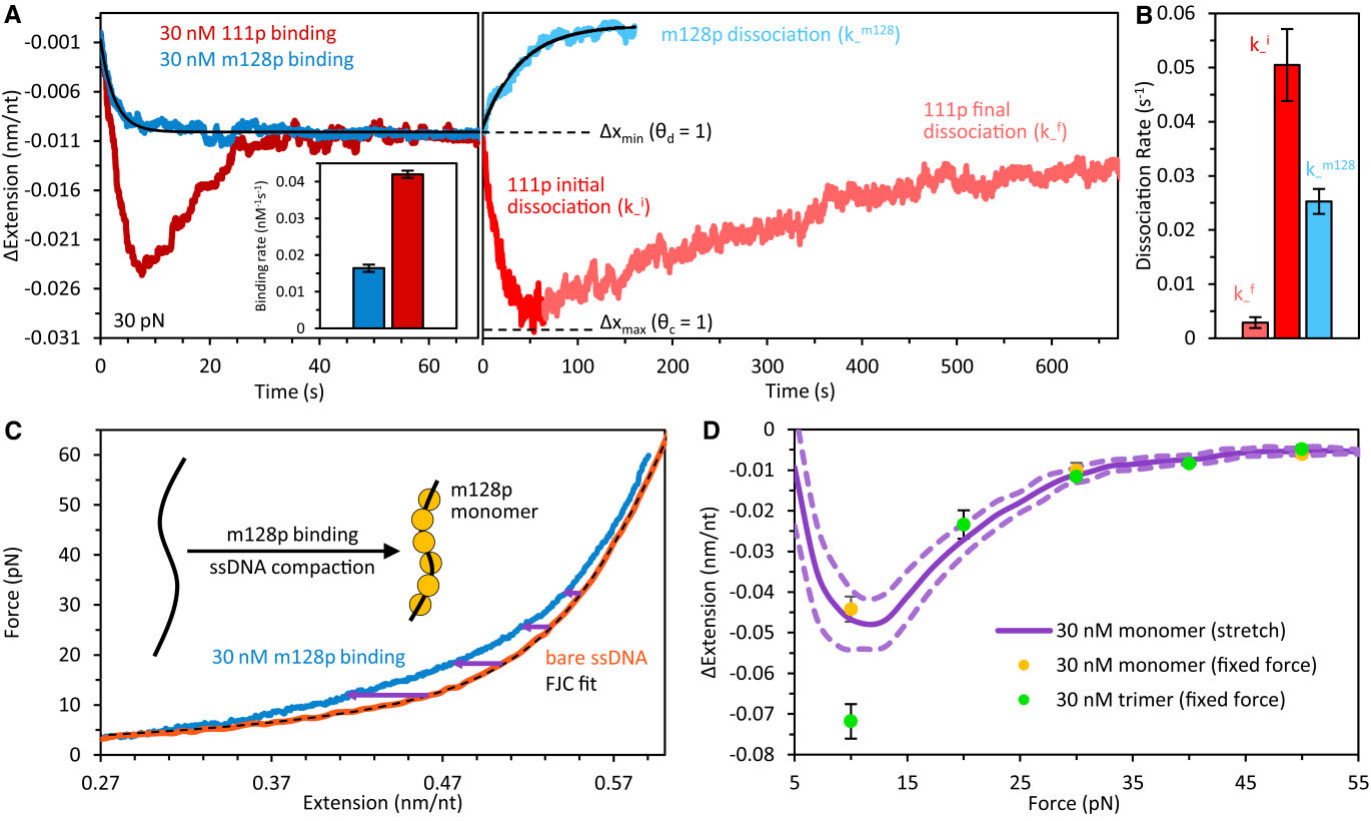
Comparison of trimeric and monomeric ORF1p binding and dissociation dynamics. (**A**) During incubation with monomeric ORF1p (m128p), the ssDNA extension decreases at a single rate (dark blue) to the same equilibrium extension (θ_d_, dashed line) as seen for high concentrations of WT ORF1p (dark red). Additionally, the bimolecular on-rate of m128p is ∼3-fold lower than that of WT (inset). In contrast to the biphasic character of WT dissociation, when free m128p is removed (light blue), the ssDNA exponentially elongates to its original length within ∼100 seconds, consistent with full dissociation of protein. (**B**) Dissociation rates of m128p and 111p are plotted for comparison. Initial dissociation of WT ORF1p from its de-compacted state (red) is over an order of magnitude (∼15-fold) faster than subsequent final dissociation from its compacted state (light red). Under identical incubation conditions, initial dissociation of trimeric 111p is ∼2-fold faster than dissociation of monomeric m128p. All dissociation data were taken with a constant protein incubation time of 100 s using 30 nM ORF1p (trimer or monomer) at 30 pN tension. (**C**) When the ssDNA is slowly stretched (∼10 nm/s) in the presence of a saturating concentration (30 nM) of m128p (blue), the ssDNA is measurably shorter than bare ssDNA (orange with dashed black line showing fit to the freely-jointed chain polymer model). The average extension change of ssDNA as a result of m128p binding (purple arrows) is calculated at every force (1 pN increments) and plotted as a function of substrate tension (**D**). The extension reduction of ssDNA measured via equilibrium stretching (purple line with dashed lines showing SEM) and constant force (yellow circles) decreases at high tension (> 15 pN) and agrees with the equilibrium extension changes from constant force incubation experiments with high WT ORF1p concentrations (green circles, Δx_+_^f^ in Figure 3F). In contrast, at lower forces (∼10 pN), m128p does not compact ssDNA to the same degree as WT ORF1p.

The m128p truncate, which contains only the CTD, RRM and 3.5 of the 14 CC heptads (residues 128–338), is primarily monomeric at 21°C and 30 nM (17). In contrast to the biphasic binding of full-length ORF1p, m128p exhibits simple on-off binding to ssDNA (Figure 5A and Supplementary Figure S3). Compaction occurs exponentially at a single rate, and upon protein removal, the DNA monotonically elongates to its protein-free length. Thus, only the trimer can convert between compact and de-compact conformations. Moreover, the maximum extension reduction by m128p is ∼3-fold less than that produced by WT ORF1p (Δx_max_), indicating that the full-length trimer is required for full compaction of DNA.

The bimolecular on-rate of monomeric ORF1p is ∼3-fold lower than that of the trimer, likely a consequence of its reduced binding surface. Notably, initial dissociation of m128p is ∼2-fold slower than that of 111p (Figure 5B and Supplementary Table S7), indicating that de-compacted trimers are more labile (dissociate more readily) than ORF1p monomers. Additionally, the equilibrium extension change (Δx_m128_) upon incubation with saturating m128p is approximately equivalent to the minimal equilibrium extension change observed with high concentrations of full-length ORF1p (Δx_min_, Figure 5A and Supplementary Figure S3C).

This equivalency holds over a range of forces. By stretching the ssDNA (∼10 nm/s to maintain equilibrium) in the presence of a saturating concentration (30 nM) of m128p, we measured the total compaction associated with protein binding as a function of force (Figure 5C and D). Comparing this result with the equilibrium extension changes due to the binding of 30 nM WT ORF1p (Δx_+_^f^, see Figure 3F) reveals that at ≥ 20 pN tension, the equilibrium compaction of the ssDNA substrate is approximately equivalent for both proteins (Figure 5D). However, given the considerably different environments of the monomeric and trimeric binding sites, it is unclear if only a single subunit of the ORF1p trimer is associating with the ssDNA substrate when in the de-compacted conformation. At low force (10 pN), the results diverge, and the WT ORF1p-ssDNA complex remains in a more compact equilibrium state subsequent to the de-compaction transition.

### ORF1p binding in the presence of oligonucleotides

In the absence of NAs, ORF1p trimers form insoluble aggregates in NA-binding buffer (50 mM Na^+^; see the ‘Materials and methods’ section). Thus, ORF1p in molar excess of NA likely exists as aggregates. However, we had previously shown that aggregated trimers are fully capable of binding NAs, and the addition of NA, including oligonucleotides as short as 20 nt, immediately resolves the aggregates into soluble trimers (17). Therefore, to determine if aggregation of ORF1p affects our binding measurements, we incubated ORF1p with an equimolar concentration of single-stranded dT_20_ before adding it to the tethered 8.1 knt ssDNA substrate. At 5 pN (low force) and 30 nM ORF1p, equimolar dT_20_ does not significantly affect protein binding to the 8.1 knt ssDNA (Supplementary Figure S4A and Supplementary Table S8). This result is consistent with previous studies, which showed that a 20-mer of ssDNA binds to ORF1p with about 1/20th the affinity of a 60- or 120-mer, and is thus readily displaced by a longer substrate (17).

**Figure 6.**
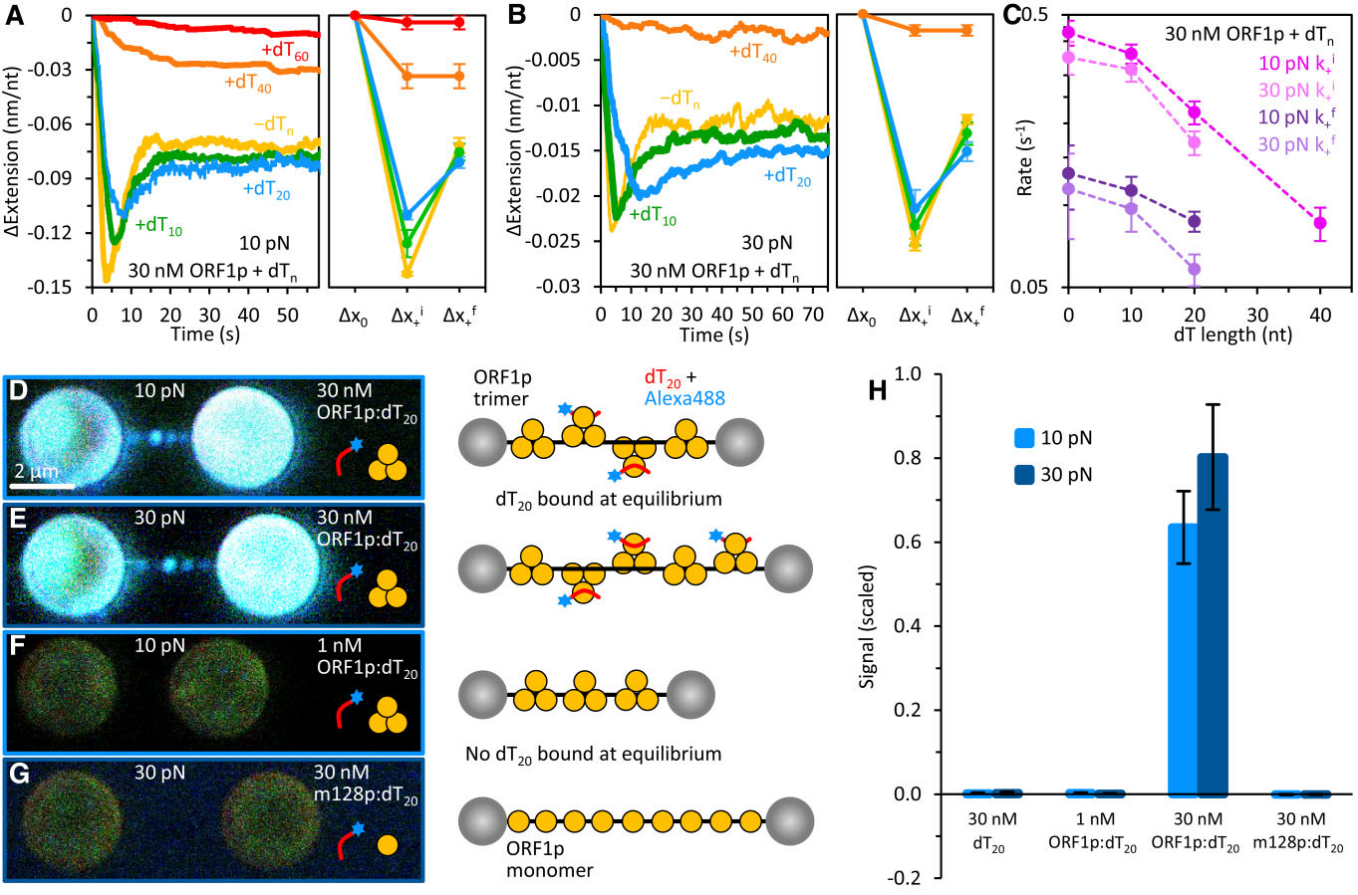
ORF1p binding in the presence of (dT_n_) oligos. Representative binding curves (left) and average ssDNA extension changes (right) following incubation with 30 nM ORF1p at 10 pN (**A**) and 30 pN (**B**) in the presence and absence of equimolar single-stranded dT_n_ oligos (n = 10, 20, 40 and 60 nt). In the presence of dT_10-20_, biphasic binding becomes somewhat less pronounced, and the complexes equilibrate to moderately more compact states. As the oligo length is increased, ssDNA compaction is significantly reduced, and the binding profiles become single-phased. (**C**) The rates of compaction and elongation decrease with the length of the dT oligos. While these rates are only slightly affected by the presence of dT_10_, we observe a significant reduction in the compaction and elongation kinetics as the oligo length is increased to n ≥ 20 nt. Following incubation at 10 pN (**D**) and 30 pN (**E**) with 30 nM ORF1p and equimolar dT_20_, end-labeled with Alexa488, we observe increased fluorescence intensity along the tethered ssDNA molecule, indicating simultaneous binding of the protein to the labeled oligo and long substrate. All images are analyzed using the same intensity scaling to ensure consistency in integrated fluorescence measurements across different conditions. Note, during the tethering process, free (8.1 knt) DNA in solution tends to aggregate and saturate the streptavidin-coated microbeads (spheres). Upon transfer into the protein-containing channel, DNA saturation can promote subsequent binding of the protein and oligos to the beads, resulting in increased fluorescence signal above typical bead autofluorescence. (**F**) Low protein concentrations (1 nM), which result in full DNA compaction (see Supplementary Figure S3B), show no fluorescence above background. (**G**) Similarly, no signal is observed following incubation with 30 nM monomeric m128p. (**H**) Integrated fluorescence signal of the 8.1 knt ssDNA in the presence of labeled dT_20_ alone, and in equimolar concentrations with the trimer and monomer as shown. Only high concentrations of trimeric ORF1p show an increase in fluorescence along the tethered ssDNA substrate. Images and signals are scaled as described in the ‘Materials and methods’ section.

However, at higher tensions (≥ 10 pN), dT_20_ does affect ORF1p binding to the 8.1 knt ssDNA. While the added oligonucleotides do not significantly alter the equilibrated conformation of the protein–DNA complex, the rates of binding (k_+_^i^ and k_+_^f^) are suppressed (Supplementary Figure S4B and Figure 6A–C). To further elucidate these dynamics, we determined the effect of oligonucleotide length on the interaction of ORF1p with the tethered DNA (Figure 6A–C and Supplementary Table S9). In equimolar dT_10-20_, initial DNA compaction (Δx_+_^i^) is moderately reduced, yet the binding profiles remain biphasic, indicating sufficient protein binding to facilitate transition to a (mostly) de-compacted state. However, in the presence of dT_40-60_, compaction is severely inhibited and the extension-time profiles become single-phased, indicating insufficient binding to saturate the 8.1 knt ssDNA. Thus, the extent (and rate) of protein binding to the tethered DNA decreases with the length of the added oligonucleotide, nearly vanishing as the oligonucleotide approaches the size of an ORF1p NA binding site (40–60 nt) (36).

Callahan *et al.* (17) previously showed that when ORF1p binds to oligonucleotides long enough to accommodate 2–3 trimers (i.e. n = 120), inter-protein interactions enhance oligonucleotide binding; the trimers are close enough to be chemically cross-linked and can hijack trimers bound to oligonucleotides that are only long enough to accommodate a single trimer (e.g. n ≤ 50 nt). Therefore, the tethered ssDNA substrate (8.1 knt) would be expected to outcompete the shorter dT_10-60_ oligos for binding to the protein. However, this did not occur (Figure 6A-B). Unlike the 8.1 knt ssDNA, the oligonucleotides are not held under tension and are thus able to accommodate (freely conform to) the ORF1p NA binding site. As tension both reduces the affinity of ORF1p for ssNA and inhibits oligomerization of NA-bound trimers, decreased binding to the 8.1 knt DNA is likely the result of the oligonucleotides progressively outcompeting the long substrate. This is consistent with further reduction in protein binding observed at higher tension (30 pN, Figure 6B) and emphasizes the role of NA flexibility to maximize its contacts with ORF1p.

We also monitored ssDNA compaction and elongation over sequential changes in protein concentration. Following equilibration of the first round of biphasic binding, unbound ORF1p was removed by replacement with ORF1p-free buffer, leading to further compaction of the DNA (Supplementary Figure S3C). Upon reaching a quasi-stable, maximally compact state, the ssDNA compaction was reversed by reintroducing protein into solution. This process occurred through repeated changes in free protein concentration, both in the absence and presence of equimolar dT_20_ (Supplementary Figure S4C and D). Thus, conversion of ORF1p trimers between compact and de-compact binding conformations is indifferent to ORF1p aggregation. We also determined the effect of dT_20_ on ORF1p dissociation upon removing free protein. Supplementary Figure S5 shows that in the presence of dT_20_, ORF1p dissociation is both faster and more complete, suggesting that the presence of additional ssNA facilitates ORF1p displacement from the 8.1 knt DNA molecule.

### Visualization of ORF1p and dT_20_ binding to ssDNA

To visualize ORF1p-NA interaction we incubated tethered ssDNA with 30 nM ORF1p:Alexa-labeled dT_20_ (see the ‘Materials and methods’ section). Figure 6D, E and H reveal that at equilibrium, fluorescence is distributed along the entire tethered ssDNA molecule. As the fluorescent oligos do not intrinsically colocalize with the long ssDNA substrate (Supplementary Figure S6), we conclude that ORF1p is simultaneously binding the fluorescent dT_20_ and tethered ssDNA. At this protein concentration, the ORF1p-ssDNA complex primarily exists in a de-compacted conformation at equilibrium (Figure 6A and B). By contrast, a fully compacted ORF1p-DNA complex (i.e. 1 nM ORF1p:dT_20_; Supplementary Figure S4B) does not bind fluorescent dT_20_ at equilibrium (Figure 6F and H). Thus, formation of a tripartite complex between the protein and two NA substrates requires de-compacted ORF1p binding conformers. We also performed these measurements with a high concentration (30 nM) of the truncated monomeric ORF1p, m128p, and did not detect any fluorescence above background along the tethered ssDNA (Figure 6G and H). Thus, only the trimer can simultaneously engage two ssNA molecules: the tethered ssDNA and Alexa-labeled dT_20_.

## Discussion

### Assembly of distinct ORF1p configurations on ssNA

The conformation of an ORF1p NP (ORF1p-NP) depends on the molar ratio of protein and ssDNA. When the amount of protein is just sufficient (several fold, but not orders of magnitude, above K_D_) to engage the entire NA molecule, ORF1p assumes a stable, tightly compacted conformation (θ_c_). However, at higher concentrations, additional ORF1p binds to the ORF1p-NP and destabilizes it, resulting in de-compaction and concomitant elongation of the ssNA. At sufficiently high protein concentration, the NP can transition to a fully de-compacted mode (θ_c_ → θ_d_, Figure 3A–D). Moreover, the ORF1p-NP is capable of repeated cycles of compaction and de-compaction upon removal of free protein and its subsequent replacement (Supplementary Figure S4C and D), suggesting that conversion between ORF1p states is a function of DNA-bound protein (i.e. protein density). The oligomerization-deficient CC mutant, 151p (Supplementary Figure S1), exhibited nearly identical biphasic binding as WT ORF1p, indicating that transition between compact and de-compact NP states is not primarily a consequence of ORF1p oligomerization (protein–protein interactions) on ssNA.

**Figure 7.**
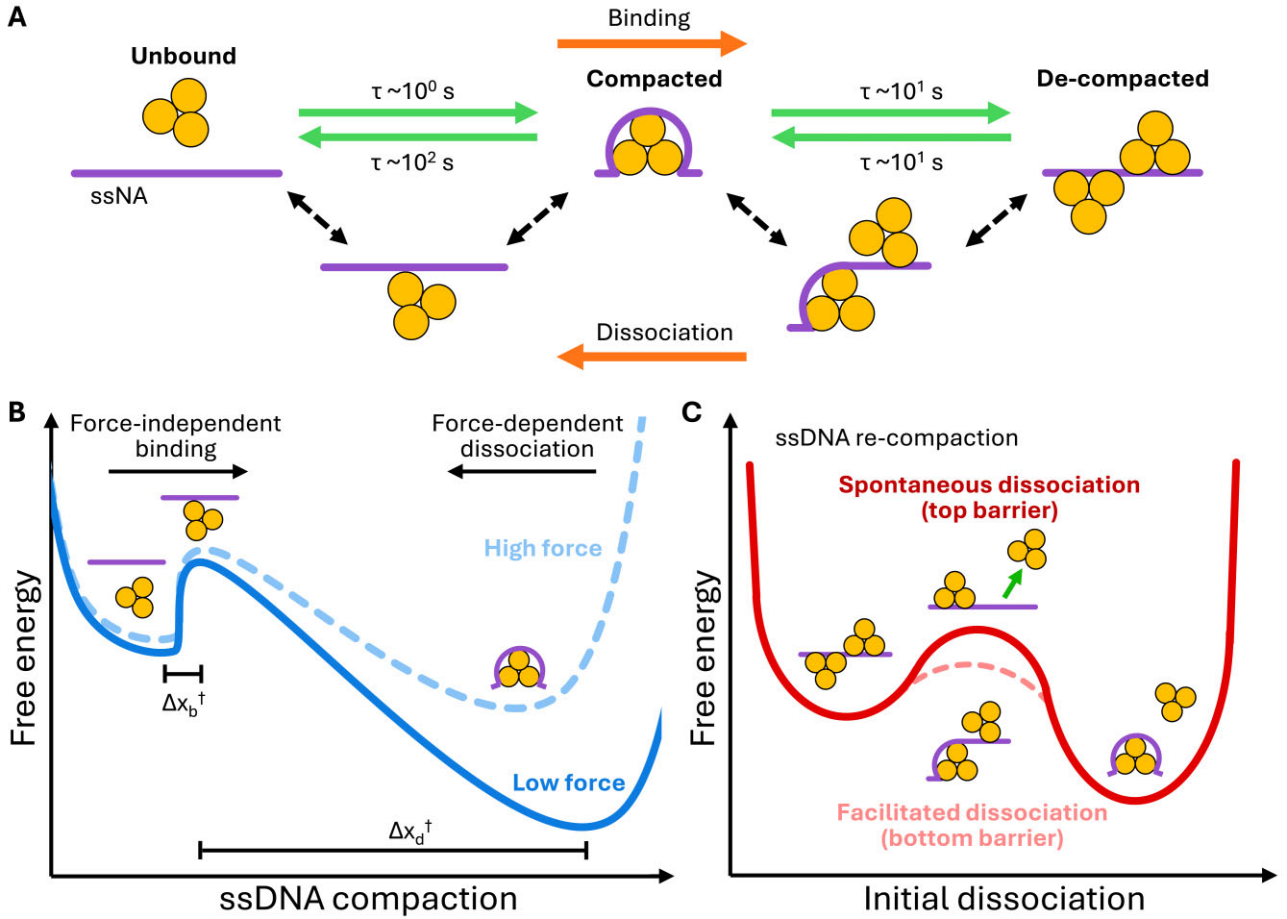
Proposed model of ORF1p binding and conformational transitions. (**A**) Upon binding, ORF1p initially compacts ssDNA, with additional protein binding producing a de-compacted (overcrowded) state as a fraction of nucleotides engaged with ORF1p are transferred to the binding site of a second trimer (top row). This process is reversed upon dissociation of ORF1p. Proposed transition states (bottom row) allow for initial binding of free protein with minimal deformation of the NA substrate, resulting in force-independent rates of NP compaction and de-compaction. (**B**) Proposed free energy landscape of ssDNA corresponding to two stable states, protein-free (left) and protein-bound (compacted, right), separated by an energy barrier whose height determines the rate of protein binding (ssDNA compaction) and dissociation (elongation). The landscape (solid line) is plotted at high protein concentration, such that the bound state is favored. Applying force shifts the energy landscape (dashed line), increasing free energy by the product of ssDNA tension and displacement (length change to the transition state, Δx^†^). The compacted state becomes less stable (increased free energy) relative to the unbound state. Our measured rates (see Figures 3G and 4F) indicate that the transition state lies much closer to the unbound state (Δx_b_^†^ << Δx_d_^†^), such that the rate of compaction is less dependent on force than the rate of dissociation. (**C**) Proposed free energy profile of ssDNA re-compaction during initial dissociation of ORF1p showing two stable states, a de-compacted state (left) and a compacted state (right). Spontaneous dissociation from the de-compacted protein state (top barrier, solid line) would leave behind regions of bare ssDNA (transiently), resulting in a greater free energy cost to transition (higher barrier). However, simultaneous incorporation of nucleotides released from departing proteins into the binding site of remaining ssDNA-bound trimers (bottom barrier, dashed line) would reduce the free energy cost to transition (smaller barrier), facilitating conversion to the fully compacted NP conformation.

Figure 6 shows that de-compacted ORF1p trimers, bound to the tethered 8.1 knt ssNA, can simultaneously engage a fluorophore-labeled ss dT_20_ oligonucleotide. Fluorescence was distributed along the entire ssDNA, indicating concurrent binding of ORF1p to both the long (8.1 knt) DNA template and Alexa-labeled dT_20_ oligonucleotide (Figure 6D, E and H). However, under conditions of full DNA compaction, no fluorescence was detected. As trimeric ORF1p can accommodate ∼50 nt (36), each trimer subunit binds ∼17 nt of ssDNA. Therefore, concurrent binding of the protein to the 20 nt oligo and long ssDNA suggests that at least one of the three trimer monomers (NA binding site) is disengaged from the tethered DNA when ORF1p is in its de-compacted conformation. Moreover, the absence of fluorescence at 1 nM ORF1p (Figure 6F) implies that each of the three NA-binding domains are engaged with the ssDNA, and therefore not available for binding to another NA substrate, when the complex is in its most compact state. Therefore, transition to the de-compacted NP state must involve partial ssDNA release from the ORF1p binding surface, likely facilitated by competition of ORF1p trimers for the ssNA substrate (see Figures 2C and 7A).

We did not obtain such results (e.g. cohabitation of ORF1p with multiple NA substrates, biphasic binding) with monomeric ORF1p, m128p (Figure 5, 6G and H, and Supplementary Figure S3). Therefore, the ability to bind NA in multiple conformations is solely a property of the trimer, consistent with structural studies showing that trimeric ORF1p possesses surface features that may allow a single continuous strand of NA to wrap around the protein's binding site through multiple paths (18).

Applying force to the DNA reduces ORF1p-mediated NA compaction (Figure 3E and F). However, the rate of initial compaction induced by protein binding (k_+_^i^) was largely insensitive to the tension on the ssDNA substrate (Figure 3G), indicating that the rate-limiting step to binding is likely diffusion-limited and does not involve significant DNA deformation. In contrast, the rate of subsequent elongation, concomitant with protein dissociation (k_−_^f^), increased dramatically with substrate tension (Figure 4F), implying that the final release of ssDNA requires considerable DNA lengthening, consistent with the dissociation of ORF1p from its maximally compact state. Overall, these rate dependencies are consistent with an energy landscape (illustrated in Figure 7B), in which the (rate-limiting) transition step lies much closer to the unbound and uncompacted protein state than to the bound and compacted conformation. Specifically, the initial ORF1p-NA binding state is not the highly compacting state observed at equilbirium, but rather a transition state in which free protein localizes onto minimally constrained ssNA, such that it only tangentially interacts with the protein binding surface. However, this does not preclude the possiblity of an additional, much faster (non-rate limiting) force-dependent compaction step subsequent to initial protein localization.

ORF1p dissociation occurs in two phases and appears to be the reversal of its biphasic binding. Upon removal of free protein, the ssDNA rapidly shortens and returns to its initial compacted length (Figure 2C and 4A, and Supplementary Figure S2A), suggesting that nucleotides released by departing proteins are incorporated into remaining ssDNA-bound trimers, enabling maximal engagement between NA and the ORF1p binding site (Figure 7A and C). Following this step, the DNA slowly elongates and approaches its protein-free conformation, indicating release of ssDNA from the fully compacted ORF1p trimers. Both phases of dissociation are sensitive to incubation conditions (e.g. time, trimer concentration and DNA tension), and likely reflect the oligomeric nature of NA-bound ORF1p. In particular, both the rate and extent of dissociation were moderately reduced with bulk protein concentration (Figure 4B, D and E), implying enhanced NP stability, similar to that seen with increased incubation time (Supplementary Figure S2). As dissociation occurs in the absence of free protein, it is typically independent of protein concentration. However, changes in ORF1p concentration may alter its oligomerization on NA, yielding differences in the protein's overall binding stability and resulting dissociation profile. Such an increase in oligomerization at higher bulk concentrations may be a result of: (i) additional ORF1p-NA binding (i.e. increased protein density), which promotes the formation of higher-order multimers on NA, and/or (ii) faster protein binding, such that the NP complex remains saturated for longer times prior to the removal of free protein (see Supplementary Figure S2 illustrating the effect of incubation time on ORF1p oligomerization).

The rate of WT (111p) dissociation increased with applied force and approached the dissociation rate typical of the weakly interacting 151p trimers (Figure 4C and F), indicating that DNA under high tension (∼50 pN) prevents the formation of inter-trimer contacts that mediate the oligomerization of NA-bound trimers. Conversely, the strong divergence of these rates (111p versus 151p) at ≤ 30 pN suggests that at low to moderate force, protein–DNA unbinding may be rate-limited by the breaking of stable inter-trimeric contacts between adjacently bound ORF1 proteins (i.e. de-oligomerization).

The two phases of ORF1p dissociation occur over significantly different timescales; the dissociation rate of de-compacted trimers (k_−_^i^) is ∼15-fold higher than that of the fully compacted proteins (k_−_^f^, Figure 5B). Rapid dissociation of the de-compacted trimer could result from ORF1p being less engaged with the DNA, and thus more prone to uncoupling from the substrate. For a protein with high binding affinity, such as ORF1p, the process of spontaneously uncoupling from its substrate and drifting into solution is likely quite slow due to the large loss in binding energy (Figure 7C, top barrier). However, simultaneous exchange of ssNA from one protein binding site to another (i.e. incorporation of nucleotides released by departing proteins into the binding surface of remaining ssDNA-bound trimers) could accelerate this process by ensuring no large, abrupt loss in binding energy (i.e. a lower free energy cost to transition). It is conceivable that de-compacted trimers competing for NA might ‘pull’ nucleotides away from their neighbors in order to maximize their engagement with the DNA, thereby facilitating removal of excess protein from the substrate. Such a process of facilitated dissociation (52) has been previously observed with other multi-modal ssNA binding proteins (49) and could explain why de-compacted trimers are significantly more labile (dissociate more rapidly) than ORF1p monomers (Figure 5B), which presumably engage ssDNA minimally through a single RRM–CTD binding site. Overall, the ability of a neighboring protein to actively incorporate the substrate left behind by a dissociating protein suggests an energy landscape in which transition from the de-compacted NP state to the fully compacted conformation (θ_d_ → θ_c_) proceeds through a lower free energy barrier (Figure 7C, bottom barrier), allowing the complex to reorganize on much shorter timescales than would be permitted through isolated dissociation events. This facilitated unbinding process could involve several steps of nucleotide exchange (i.e. peeling from one protein binding site into another), mechanically analogous to the simultaneous unwinding and winding of adjacent spools in a cassette tape. Taken together, our results form a comprehensive model of ORF1p’s multi-step binding and dissociation dynamics (illustrated in Figure 7A), revealing features of the ORF1p NP that may be related to its function during L1 replication, as discussed below.

### NA chaperone activity of ORF1p

**Figure 8.**
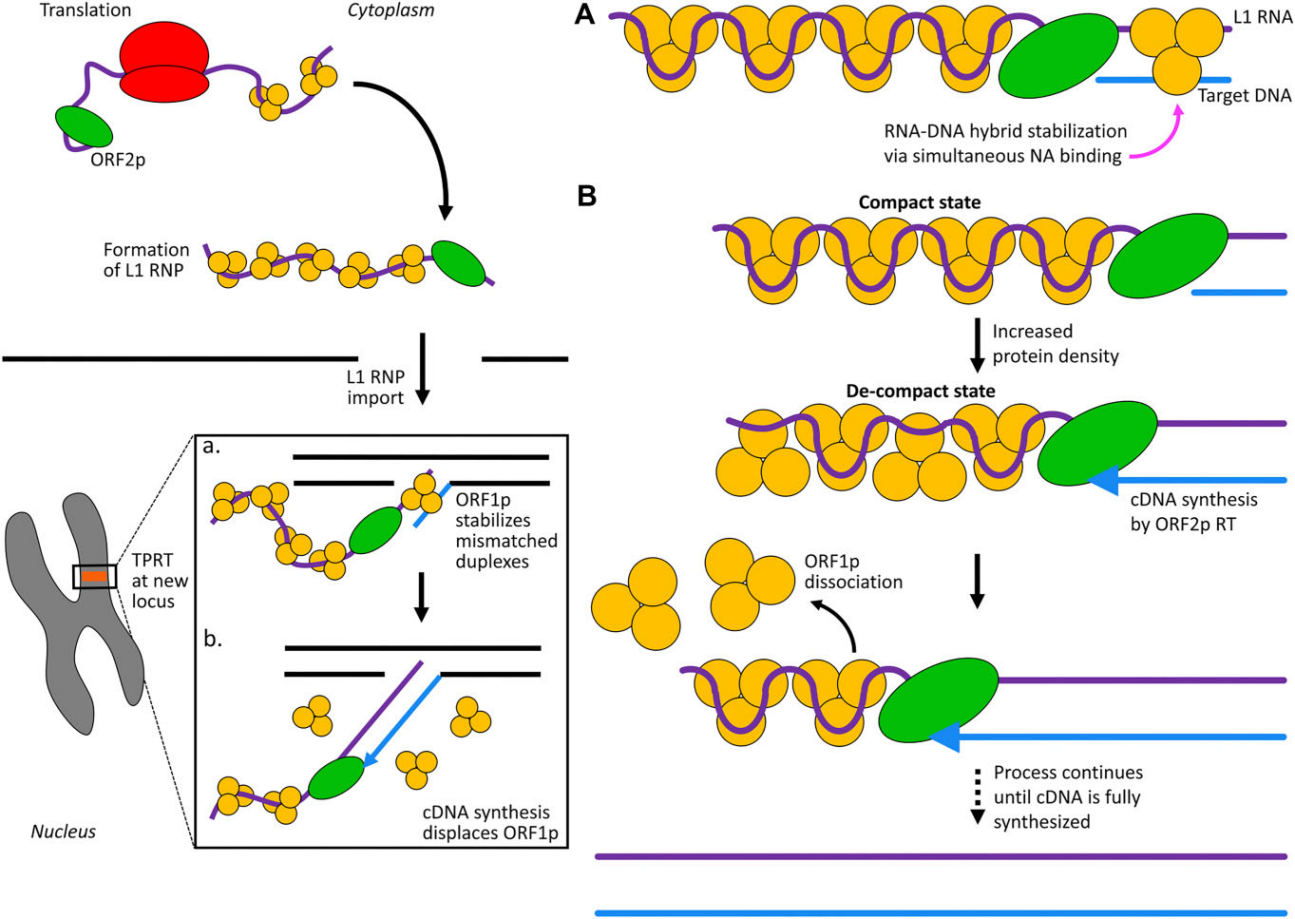
ORF1p function during TPRT. A portion of the L1 life cycle is shown on the left. Upon translation, ORF1p trimers and ORF2p assemble on their encoding transcript (*cis* preference) to form the L1 RNP. Following nuclear import, TPRT is initiated at a new locus. The bottom strand of the genomic target site (blue) is nicked by ORF2p EN and the 3′ end of the L1 RNA (purple) is annealed to the DNA flap to generate a primer for cDNA synthesis (**A**). Stable hybridization of the host DNA and L1 transcript could occur via simultaneous binding of the NA strands to different regions of the ORF1p trimer as depicted in the top right panel. Subsequently, ORF1p must be displaced from the L1 transcript during reverse transcription by ORF2p RT to synthesize the first L1 cDNA (**B**). A proposed mechanism of ORF1p removal via changes in its binding conformation is shown in the middle right panel. ORF1p is initially bound in a stable, tightly compacted state. DNA synthesis by ORF2p drives an increase in protein density as the ssNA segment shortens, forcing ORF1p into a less stable, de-compacted conformation. De-compacted trimers dissociate rapidly across the entire ssNA template, clearing the way for ORF2p. This process continues until cDNA synthesis is complete.

The NA chaperone activity of ORF1p is correlated with retrotransposition (14,33,34), indicating that one essential role of ORF1p is the facilitation of NA strand exchange during formation of a productive cDNA primer for reverse transcription (Figure 1C). We previously showed that ORF1p trimers can bind and stabilize mismatched oligonucleotide duplexes *in vitro* at 37°C, a situation likely to be encountered *in vivo* during hybridization of host DNA to the A-rich 3′ end of the L1 transcript (17,41). This process was relatively insensitive to the molar ratio of protein and NA, indicating that distinct ssNA molecules can be bound by a given trimer (i.e. an intra-trimer phenomenon). Our present biophysical data are consistent with these observations, showing that de-compacted ORF1p trimers can simultaneously engage more than one species of ssNA, a property not observed with monomeric m128p (Figure 6D–H). That the distinct ssNA binding modes of ORF1p appear to support its NA chaperone activity provides a biophysical rationale for the protein's unique trimeric structure (Figure 8).

### Dissolution of the L1 RNP during DNA synthesis

Non-specific, high affinity binding by ORF1p to ssNA would permit efficient coating of the L1 transcript, protecting it from enzymatic degradation and ameliorating secondary structure that could interfere with reverse transcription. ORF1p NPs are highly stable and resistant to dissociation (40,41). While such tight binding could benefit nuclear import by minimizing loss of protein, it could also prevent ORF1p displacement during TPRT. However, we showed that distinct conformers of ORF1p NPs (compact and de-compact), differing in affinity for ssNA, can co-exist on a given ssNA and are inter-convertible depending on their content of ORF1p, capable of inter-conformer transfer (Figure 4 and illustrated in Figure 8). These results provide a plausible mechanism for the rapid removal of ORF1p during TPRT. Compact and de-compact NPs exhibit distinct dissociation profiles, in that de-compacted ORF1p trimers dissociate ∼15-fold faster than compact ones in the absence of free protein. These results are consistent with the idea that the de-compacted conformation could serve as a transition state to dissociation, facilitating ORF1p release from NA.

Transition to a de-compacted NP state is driven by increasing ORF1p content of the ORF1p–ssNA NP (Figure 2). This condition could arise during TPRT; as ORF2p traverses the L1 RNA transcript, it shortens the template, driving the remaining ORF1p–ssNA into an overcrowded state, facilitating its conversion to a de-compacted (partially disengaged) and less stable binding mode (illustrated in Figure 8). Dissociation of ORF1p from its de-compacted binding mode is exponential, meaning that ORF1p overcrowding on the template strand can be relieved by dissociation of protein from across the entire ssNA. This mode of ORF1p removal during cDNA synthesis could enable sufficiently fast protein displacement to keep pace with the progress of ORF2p RT on the L1 RNA template while maintaining maximal coverage and protection of the uncopied L1 transcript. We observed similar dynamics (i.e. fast, exponential protein displacement upon overcrowding) with other single-stranded binding proteins, suggesting a generalizable mechanism of rapid template clearing during DNA replication (47,49).

We previously found (40,41) that oligomerization of ssNA-bound trimers retards their dissociation from ssNA, consistent with our current findings. With time, oligomerization produced an increasing amount of non-dissociable ORF1p on the timescale (∼10^3^ s) of the experiment (i.e. the ssDNA did not return to its protein-free conformation, Figure 4D). NA-bound oligomer formation is mediated by trimer–trimer interactions and is positively associated with retrotransposition competence (40), suggesting a role for oligomerization in L1 RNP assembly (tight binding and compaction), which could impede the removal of ORF1p from its ssNA template. However, we found that de-compacted NA-bound trimers are considerably weaker and more labile, dissociating faster and more completely than fully compacted trimers, regardless of the degree of protein oligomerization (Figure 4A and B, and Supplementary Figure S2). Therefore, oligomerized ORF1p could be displaced from ssNA via its conversion to a de-compacted state, which would occur with increased protein density. DNA tension can also disfavor ORF1p binding (Figure 4F), but intracellular processes typically produce forces in the range of ∼1–5 pN (53), likely too low to significantly disrupt the stable inter-trimer contacts of ORF1p. However, previous single molecule DNA stretching experiments showed that polymerases can generate tension on ssNA templates as high as ∼15–35 pN during synthesis of the complementary strand (54–58), which could be sufficient to destabilize ORF1p oligomers. The potential of ORF2p to generate such force on the L1 template, coupled with the ability of ORF1p to convert between binding modes, could provide a mechanism for dissolution of the tightly compacted L1 RNP during retrotransposition. However, if such conditions are not sufficient to promote prompt displacement of ORF1p from its template, stably bound trimers could become effective ‘roadblocks’ during TPRT, leading to ORF2p stalling and premature termination of reverse transcription, contributing to the formation of 5′ truncated L1 copies, which comprise at least 2/3 of the currently active L1Pa1 (L1Hs) family in humans (59,60).

### Retrotransposition of non-L1 transcripts

Retrotransposition of non-L1 transcripts poses two questions: How do non-L1 3′ termini generate successful primers for TPRT? How do non-L1 transcripts gain access to (hijack) the L1 RNP, which mediates retrotransposition?

Despite *cis* preference (21–24), nearly half of the genomic DNA generated by L1 activity has originated from non-L1 transcripts. Most were derived from processed pseudogenes or non-autonomous transposable elements, such as Alu and SVA (1–9,61). The 3′ termini of these transcripts mimic L1 transcripts in that they are either poly A or A-rich (5–9,62,63), features that are both necessary and sufficient for retrotransposition by L1-ORF2p (which has EN and RT activities), even in the absence of ORF1p (64).

ORF1p has several properties that could enable the retrotransposition of non-L1 transcripts. ORF1p trimers can stabilize mismatched duplexes (17), which could enable the formation of a productive RT primer complex for TPRT between non-L1 transcripts and genomic DNA, and thereby facilitate their retrotransposition.

While it is conceivable that non-L1 transcripts can compete with cytoplasmic L1 transcripts for incorporation into the L1 RNP, the proximity of ORF1p to its encoding transcript (following translation), would strongly favor the incorporation of the L1 transcript. Our current findings suggest an additional mechanism by which non-L1 transcripts can hijack the L1 RNP. ORF1p trimers assembled on an 8.1 knt ssNA produce a NP that is highly dynamic. Binding of additional ORF1p to the saturated protein–DNA complex produces a less compact and less stable NP that can accommodate more than one NA substrate. We suggest that an analogous process could occur *in vivo*. As ORF1p is displaced from its parent transcript during TPRT, it could bind and destabilize remaining downstream L1 RNP. Subsequent conversion to a de-compacted (partially disengaged state, as illustrated in Figure 8) state could allow ORF1p to engage non-L1 transcripts in the vicinity of the NP complex, making it susceptible to hijacking, and thereby enable retrotransposition of non-L1 transcripts. While the exact details of this process remain unclear, such a mechanism could explain how L1 has generated almost as much non-L1 DNA as L1 DNA, accounting for its major impact on the human genome.

As mentioned above, despite the requirement of ORF1p for TPRT-mediated L1 RNA retrotransposition, this process can also occur for some RNAs, e.g. Alu, in the absence of ORF1p, at least in cell culture-based retrotransposition assays (7,64). The Alu transcript is far shorter (∼0.3 knt) than the ∼6 knt L1 transcript (62). As ORF1p binding could both protect transcripts from degradation and ensure their suitability for reverse transcription, carrying out these functions in the absence of ORF1p would be far more challenging for L1 than Alu. Another critical aspect of ORF1p function is that its ability to support L1 retrotransposition is exquisitely sensitive to the amino acid sequence of its CC domain, which itself does not contain NA binding motifs. Thus, the conformational relationship between the three NA binding sites present in the trimer, which are likely determined by the CC, must play an important role in facilitating reverse transcription of the L1 transcript.

## Supplementary Material

gkae1141_Supplemental_File

## Data Availability

The experimental data sets are either included in the main text, supplementary material or are available from the authors upon request.
