# Supplementary material for: L1-ORF1p nucleoprotein can rapidly assume distinct conformations and simultaneously bind more than one nucleic acid
Source: Nucleic Acids Res. 2024 Nov 20;52(22):14013–29. doi: 10.1093/nar/gkae1141 (PMC11662928; doi:10.1093/nar/gkae1141)
Supplement: gkae1141_Supplemental_File [file gkae1141_supplemental_file.pdf]

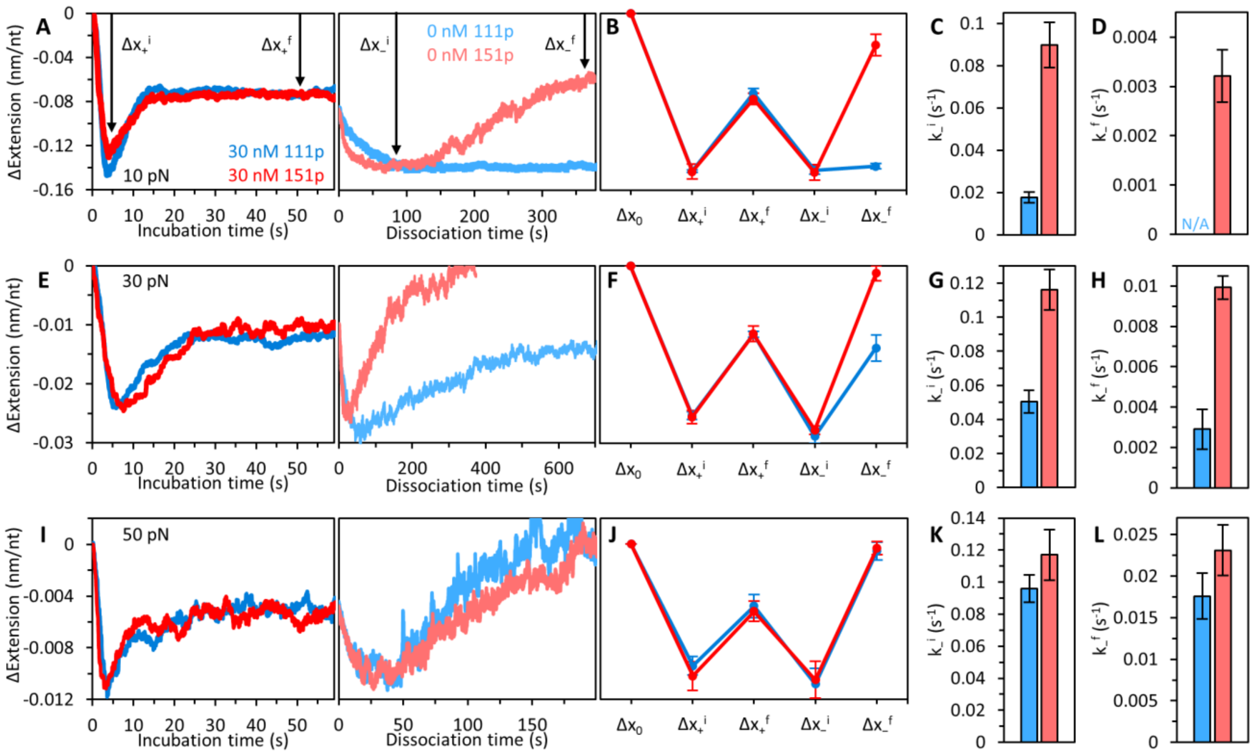

Figure S1: Comparison of WT (111) and 151 ORF1p binding and dissociation dynamics. (A-B) Initial compaction (Δx_+^i) and subsequent elongation (Δx_+^f) of the ssDNA during 151p binding (red) is nearly identical to that observed with WT ORF1p (blue) at 10 pN. However, 151p dissociation (light red) is both more complete (B, Δx_-^f) and faster (C-D) than WT (light blue). When free protein is rinsed out of solution, WT ORF1p exhibits negligible dissociation following re-compaction, while 151p dissociates nearly fully from the ssDNA (B, Δx_-^f). N/A indicates that a final dissociation rate was unattainable for WT ORF1p due to negligible dissociation on the timescale of the experiment. Similarly, at 30 pN (E-F) the characteristic biphasic binding is nearly equivalent for both proteins. The initial re-compaction (G) and final dissociation (H) phases are significantly faster for the 151p-DNA complex. (I-L) In addition to identical binding signatures, at 50 pN the two proteins exhibit similar dissociation dynamics, both in extent and rate.

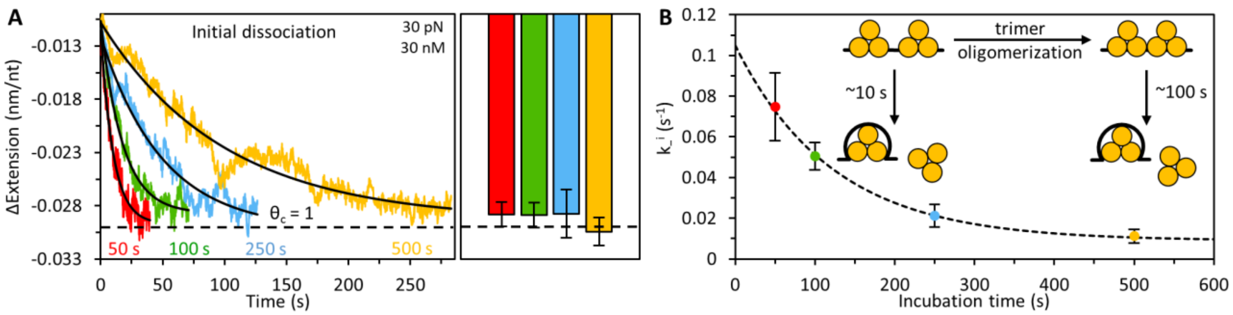

Figure S2: Incubation time dependence of initial ORF1p dissociation. (A) The ssDNA was incubated with 30 nM ORF1p at 30 pN for varying times. The protein solution was then exchanged with protein-free buffer. Increased ORF1p incubation time results in slower re-compaction of the complex during the initial dissociation phase due to enhanced protein oligomerization. Regardless of incubation time, the protein-DNA complex attains the same maximally compacted state (θ_c, dashed line). Average extension changes during initial dissociation are shown as bar plots for comparison. (B) In the limit of long incubation time, ORF1p oligomerization slows the process of re-compaction by ~10-fold. A single decaying exponential fit (dashed line) yields an oligomerization timescale of ~100 seconds under these conditions.

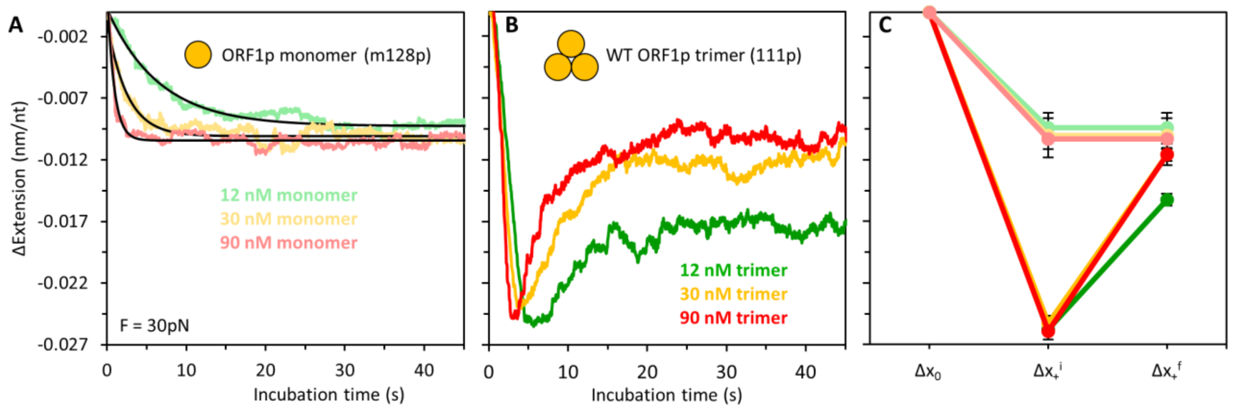

Figure S3: Comparison of trimeric and monomeric ORF1p binding. Representative binding curves of the ORF1p monomer (m128p, A) and full-length trimer (111p, B) with respect to protein concentration at 30 pN tension. m128p compacts ssDNA in a single phase, reaching the same equilibrium length at a rate proportional to protein concentration. In contrast, WT ORF1p exhibits biphasic binding, characteristic of conversion between nucleoprotein conformations. (C) Average ssDNA extension changes associated with m128p (light colors) and 111p (dark colors) binding. During incubation with monomeric ORF1p, the ssDNA extension decreases to the same equilibrium extension as seen for high concentrations of WT ORF1p.

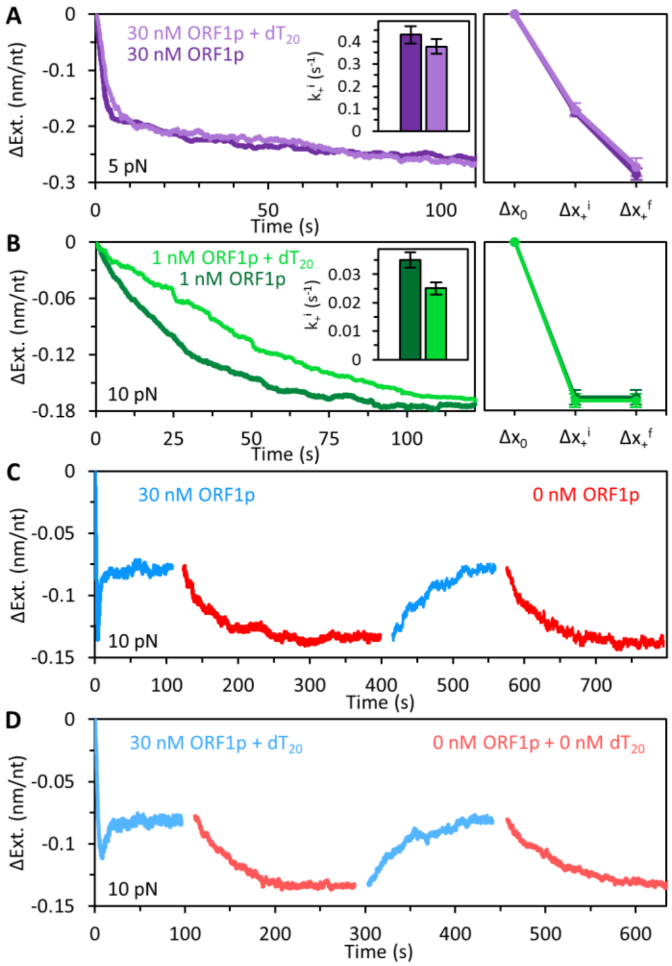

Figure S4: ORF1p binding in the presence of dT_{20} oligos. ORF1p binding to the 8.1 knt ssDNA substrate in the presence and absence of an equimolar concentration of dT_{20} oligos. (A) Representative binding curves (left, inset shows the rate of initial DNA compaction) and average extension changes (right) during incubation at 5 pN. In the presence of dT_{20}, binding to the 8.1 knt DNA is nearly identical, exhibiting rapid substrate compaction followed by a slower secondary compaction phase (see ref. 40 of the main paper). (B) Representative binding curves (left, inset shows the rate of DNA compaction) and average extension changes (right) during incubation at 10 pN with 1 nM ORF1p in the presence (light green) and absence (dark green) of equimolar dT_{20} oligos. At this concentration, both complexes exhibit single-phased compaction and equilibrate to the same final (fully compact) extension. However, the rate of DNA compaction (inset) is reduced in the presence of the oligos. In the absence (C) and presence (D) of equimolar dT_{20}, the ORF1p-ssDNA complex exhibits similar cycles of compaction and de-compaction upon removal of free protein and its subsequent replacement.

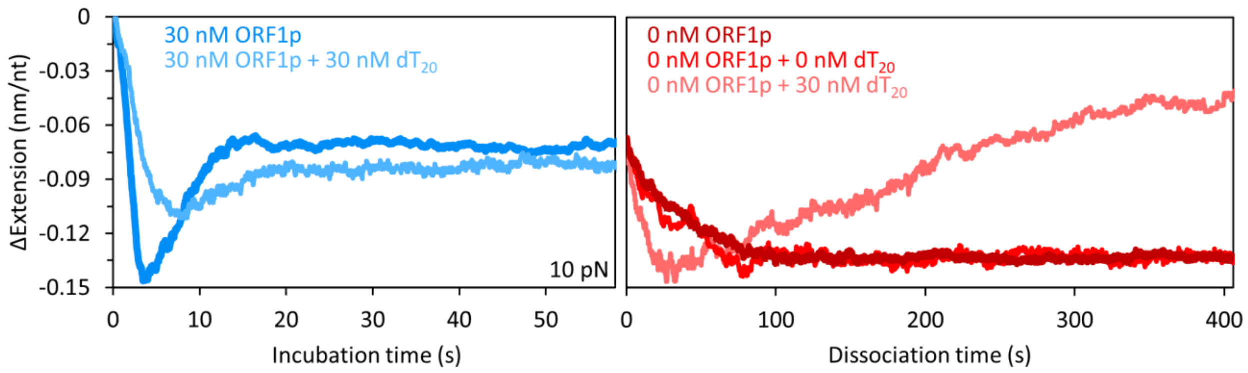

Figure S5: ORF1p binding and dissociation in the presence and absence of equimolar dT_{20}. Representative binding (left, replotted from Fig. 6A) and dissociation (right) curves at 10 pN in the presence and absence of equimolar dT_{20}. In the presence of dT_{20}, the biphasic binding signature (light blue) is less pronounced, and the complex equilibrates to a slightly more compact state. When the ORF1p-dT_{20} solution is rinsed out of the channel (red), the complex exhibits similar re-compaction as it transitions to a maximally compact state. When ORF1p is rinsed out but the oligos are kept in solution (light red), protein dissociation is both faster and more complete.

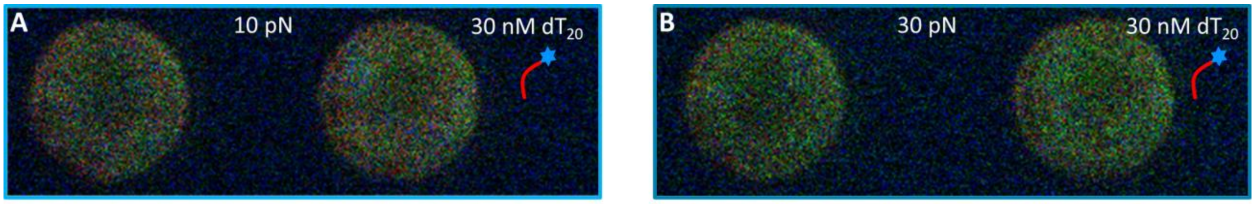

Figure S6: Confocal scans of the tethered 8.1 knt ssDNA at 10 pN (A) and 30 pN (B) in the presence of 30 nM dT_{20}, end-labeled with Alexa488. ssDNA templates exposed to dT_{20} only (no protein) show no fluorescence above background, indicating that the oligos do not intrinsically colocalize with the long ssDNA substrate (see Fig. 6H).

[ORF1p] (nM)	Δx_+^i (nm/nt)	k_+^i (s^{-1})	Δx_+^f (nm/nt)	k_+^f (s^{-1})	Θ_d
1	-0.029±0.0017	0.042±0.0087	-0.026±0.0024	N/A	0.26±0.11
3	-0.027±0.0004	0.086±0.0237	-0.020±0.0004	0.030±0.0041	0.53±0.02
12	-0.026±0.0003	0.230±0.0350	-0.015±0.0005	0.074±0.0217	0.76±0.02
30	-0.025±0.0007	0.349±0.0481	-0.012±0.0005	0.115±0.0398	0.93±0.02
90	-0.026±0.0007	0.385±0.0676	-0.012±0.0009	0.134±0.0129	0.93±0.04
Binding fits	k_b ($nM^{-1}s^{-1}$)	k_c (s^{-1})	k_b* ($nM^{-1}s^{-1}$)	k_{-c} (s^{-1})	K* (nM)
	0.042±0.0057	0.447±0.0471	0.012±0.0012	0.152±0.0128	2.8±0.32

Table S1: Concentration-dependent binding measurements. Extension changes and rates associated with each ORF1p binding phase as functions of protein concentration (see Fig. 3). All data were taken at 30 pN tension. The fractional occupancies of ssDNA-bound ORF1p trimers in the de-compacted state (θ_d) were calculated as $\theta_d = (\Delta x_{max} - \Delta x_+^f)/(\Delta x_{max} - \Delta x_{min})$, where Δx_{max} = -0.030±0.0008 nm/nt corresponds to ORF1p trimers in the fully compact state at 30 pN, and Δx_{min} = -0.010±0.0009 nm/nt corresponds to trimers in the de-compacted state. K* indicates the concentration at which both states are equally occupied. ORF1p binding and compacting rates were calculated by fitting k_+^i to a two-rate equation: $k_+^i = [1/(ck_b) + 1/k_c]^{-1}$ where c is the protein concentration, k_b is the bimolecular binding rate, and k_c is the compacting rate. The binding rate after protein saturation, k_b*, and the ORF1p de-compaction rate, k_{-c}, were calculated by fitting k_+^f to $k_+^f = [1/(ck_b^*) + 1/k_{-c}]^{-1}$. Error values are standard error of the mean or uncertainty in fitting parameters based on $\chi^2 + 1$. N/A indicates that the transition rate, k_+^f, was unattainable due to lack of biphasic binding at 1 nM ORF1p.

Force (pN)	Δx_+^i (nm/nt)	k_+^i (s^{-1})	Δx_+^f (nm/nt)	k_+^f (s^{-1})
5	-0.176±0.0044	0.430±0.0379	-0.286±0.0103	0.009±0.0013
10	-0.143±0.0011	0.431±0.0453	-0.072±0.0043	0.131±0.0347
20	-0.044±0.0037	0.335±0.0759	-0.023±0.0035	0.073±0.0137
30	-0.025±0.0007	0.349±0.0481	-0.012±0.0005	0.115±0.0398
40	-0.016±0.0007	0.434±0.0843	-0.008±0.0006	0.084±0.0187
50	-0.009±0.0007	0.424±0.0504	-0.005±0.0009	0.090±0.0102
Binding fits	N/A	0.407±0.0100	N/A	0.101±0.0070

Table S2: Force-dependent binding measurements. Extension changes and rates associated with each binding phase as functions of ssDNA tension (see Fig. 3). All data were taken with 30 nM ORF1p. Both the bind-compact and bind-decompact transition rates are independent of template tension. The force-independent values of k_+^i and k_+^f were calculated by fitting a straight line to the data (Fig 3G). k_+^f at 5 pN denotes the rate associated with secondary compaction as discussed in ref. 40 of the main paper.

	F = 10 pN		F = 30 pN		F = 50 pN	
[ORF1p] = 30 nM	151p	111p	151p	111p	151p	111p
Δx_+^i (nm/nt)	-0.143±0.0067	-0.143±0.0011	-0.026±0.0011	-0.025±0.0007	-0.010±0.0011	-0.009±0.0007
k_+^i (s^{-1})	0.422±0.0176	0.431±0.0453	0.371±0.0471	0.349±0.0481	0.416±0.0389	0.424±0.0504
Δx_+^f (nm/nt)	-0.077±0.0051	-0.072±0.0043	-0.012±0.0013	-0.012±0.0005	-0.005±0.0008	-0.005±0.0009
k_+^f (s^{-1})	0.126±0.0279	0.131±0.0347	0.135±0.0141	0.121±0.0398	0.124±0.0255	0.090±0.0102
Δx_-^i (nm/nt)	-0.144±0.0069	-0.142±0.0038	-0.028±0.0007	-0.029±0.0012	-0.011±0.0014	-0.011±0.0012
k_-^i (s^{-1})	0.090±0.0108	0.018±0.0025	0.116±0.0118	0.050±0.0067	0.117±0.0158	0.096±0.0087
Δx_-^f (nm/nt)	-0.028±0.0099	-0.138±0.0019	-0.001±0.0013	-0.014±0.0022	-0.0003±0.0005	-0.0005±0.0007
k_-^f (s^{-1})	0.003±0.0005	<0.001	0.010±0.0006	0.003±0.0010	0.023±0.0031	0.018±0.0027

Table S3: Comparison of WT (111) and 151 ORF1p binding. Extension changes and rates associated with each binding and dissociation step as functions of ssDNA tension for WT ORF1p (111p) and 151p (see Fig. S1). All data were taken with a protein incubation concentration of 30 nM and an incubation time of 100 s.

Incubation time (s)	Δx_-^i (nm/nt)	k_-^i (s^{-1})
50	-0.029±0.0012	0.075±0.0167
100	-0.029±0.0012	0.051±0.0067
250	-0.029±0.0023	0.021±0.0055
500	-0.030±0.0013	0.011±0.0033
k_{oligo} (s^{-1})	$k_-^i(t = 0)$ (s^{-1})	$k_-^i(t \rightarrow \infty)$ (s^{-1})
0.009±0.0030	0.108±0.0258	0.009±0.0036

Table S4: Incubation time dependence of initial dissociation. Extension changes and rates associated with initial ORF1p dissociation as functions of incubation time (see Fig. S2). All data were taken at 30 pN with an incubation concentration of 30 nM. The initial dissociation rate, k_-^i, was fit with a single decaying exponential to compute the ORF1p oligomerization rate (k_{oligo}) at 30 pN, as well as the limiting values ($k_-^i(t = 0)$ and $k_-^i(t \rightarrow \infty)$) of the ORF1p initial dissociation rate.

[ORF1p] (nM)	Δx_{-}^{i} (nm/nt)	k_{-}^{i} (s^{-1})	Δx_{-}^{f} (nm/nt)	k_{-}^{f} (s^{-1})
1	-0.029±0.0015	N/A	-0.001±0.0008	0.007±0.0007
3	-0.028±0.0008	0.090±0.0124	-0.003±0.0016	0.006±0.0006
12	-0.028±0.0010	0.065±0.0028	-0.013±0.0015	0.004±0.0004
30	-0.029±0.0012	0.050±0.0067	-0.016±0.0019	0.003±0.0010
90	-0.030±0.0017	0.046±0.0055	N/A	N/A

Table S5: Concentration-dependent dissociation measurements. Extension changes and rates associated with the initial and final ORF1p dissociation phases as functions of protein concentration (see Fig. 4). All data were taken at 30 pN tension with an incubation time of 100 s. N/A indicates lack of re-compaction transition at 1 nM ORF1p or data not taken.

Force (pN)	30 nM [ORF1p] incubation Δx_-^i (nm/nt)	k_-^i (s^{-1})	Δx_-^f (nm/nt)	k_-^f (s^{-1})	1 nM [ORF1p] incubation Δx_-^f (nm/nt)	k_-^f (s^{-1})
10	-0.142±0.0038	0.018±0.0025	-0.138±0.0019	<0.001	-0.0006±0.0018	0.002±0.0003
20	-0.042±0.0058	0.038±0.0069	N/A	N/A	N/A	N/A
30	-0.029±0.0012	0.050±0.0067	-0.014±0.0022	0.003±0.0010	-0.0013±0.0007	0.007±0.0007
40	-0.015±0.0012	0.086±0.0273	N/A	N/A	N/A	N/A
50	-0.011±0.0012	0.096±0.0087	-0.0005±0.0007	0.018±0.0027	-0.0002±0.0002	0.020±0.0038

Table S6: Force-dependent dissociation measurements. Extension changes and rates associated with the initial and final ORF1p dissociation phases as functions of ssDNA tension (see Fig. 4). Initial dissociation measurements (Δx_-^i and k_-^i) were taken with a protein incubation concentration of 30 nM. Final dissociation measurements (Δx_-^f and k_-^f) were taken with incubation concentrations of 30 nM (left) and 1 nM (right). All data were taken with an incubation time of 100 s. N/A indicates data not taken.

Δx_{m128} (nm/nt)	k_{obs} (s^{-1})	k_{on} ($nM^{-1}s^{-1}$)	k_{off} (s^{-1})	k_{off}/k_{on} (nM)
-0.010±0.0018	0.492±0.0299	0.016±0.0010	0.025±0.0037	1.6±0.18

Table S7: Monomeric m128p binding and dissociation measurements. Extension change and rates associated with m128p binding and dissociation (see Fig. 5). Binding data were taken at 30 pN with 30 nM m128p. The on-rate was calculated as $k_{on} = (k_{obs} – k_{off})/c$ with k_{obs} and k_{off} computed by fitting the binding and dissociation data to single exponentials, respectively.

n = 20 nt	F = 5 pN, [ORF1p] = 30 nM			F = 10 pN, [ORF1p] = 1 nM		
	Δx_+^i (nm/nt)	k_+^i (s^{-1})	Δx_+^f (nm/nt)	Δx_+^i (nm/nt)	k_+^i (s^{-1})	Δx_+^f (nm/nt)
w/out dT_n	-0.176±0.0044	0.430±0.0379	-0.286±0.0103	-0.166±0.0079	0.035±0.0027	-0.166±0.0079
w/ dT_n	-0.171±0.0121	0.378±0.0316	-0.272±0.0159	-0.170±0.0073	0.025±0.0022	-0.170±0.0073

Table S8: ORF1p binding in the presence and absence of equimolar dT_{20} oligos. Extension changes and rates associated with ssDNA compaction during incubation with ORF1p in the presence and absence of an equimolar concentration of dT_{20} (see Fig. S4).

dT length (nt)	F = 10 pN				F = 30 pN			
	Δx_+^i (nm/nt)	k_+^i (s^{-1})	Δx_+^f (nm/nt)	k_+^f (s^{-1})	Δx_+^i (nm/nt)	k_+^i (s^{-1})	Δx_+^f (nm/nt)	k_+^f (s^{-1})
No oligos	-0.143 ±0.0011	0.431 ±0.0453	-0.072 ±0.0043	0.131 ±0.0347	-0.025 ±0.0007	0.349 ±0.0481	-0.012 ±0.0005	0.115 ±0.0398
n = 10	-0.126 ±0.0075	0.360 ±0.0290	-0.075 ±0.0046	0.113 ±0.0138	-0.023 ±0.0021	0.316 ±0.0314	-0.013 ±0.0012	0.097 ±0.0172
n = 20	-0.110 ±0.0025	0.219 ±0.0212	-0.081 ±0.0031	0.087 ±0.0074	-0.021 ±0.0021	0.170 ±0.0170	-0.015 ±0.0011	0.058 ±0.0078
n = 40	-0.034 ±0.0067	0.086 ±0.0121	-0.034 ±0.0067	N/A	-0.002 ±0.0006	N/A	-0.002 ±0.0006	N/A
n = 60	-0.004 ±0.0038	N/A	-0.004 ±0.0038	N/A	N/A	N/A	N/A	N/A

Table S9: ORF1p binding in the presence of dT oligos. Extension changes and rates associated with ORF1p binding in the presence and absence of single-stranded dT_n oligos (see Fig. 6). All data were taken with 30 nM ORF1p. N/A indicates rates or extension changes that were unattainable due to lack of binding or negligible DNA compaction.
